# Supplementary material for: In silico unwinding of Caenorhabditis elegans microRNA duplexes to evaluate thermodynamic end stabilities improves predictions of microRNA strand selection
Source: RNA Biol. 2026 Mar 24;23(1):1–18. doi: 10.1080/15476286.2026.2649359 (PMC13048549; doi:10.1080/15476286.2026.2649359)
Supplement: Medley_Supplemental_Revised.pdf [file KRNB_A_2649359_SM8245.pdf]

Supplemental Data

In silico unwinding of *Caenorhabditis elegans* microRNA duplexes to evaluate thermodynamic end stabilities improves predictions of microRNA strand selection

Jeffrey C. Medley[1] and Anna Zinovyeva[1,2]

[1]Division of Biology, Kansas State University. Manhattan, KS, USA.

[2]Correspondance: zinovyeva@ksu.edu

Figure S1: Comparison of structural information obtained from central miRNA repositories. (A) Comparison of the (A) *let-7*, (B) *lin-4*, (C), *mir-1*, (D) *mir-2* and (E) mir-35 hairpin structures from miRBase v22.1 (left) and MirGeneDB 3.0 (right). (B) Structure of the lin-4 hairpin obtained from miRBase (left) and MirGeneDB (right). For miRBase structures, nucleotides contained within the duplex structure are given as pink, uppercase text. For MirGeneDB structures, guide strands are given as red text and passenger strands are indicated by blue text. Yellow highlighting indicates locations of motifs involved in miRNA processing.

Figure S2: Quantification of temperature-dependent predictions of (A) miRNA hairpin and (B) miRNA duplex folding relative to default temperature parameters (37ºC). (A-B) The location of alternative folding is indicated by different colors. The number of observed differences is given at the bottom of each bar (n= 253 miRNA hairpins and n=190 miRNA duplexes). Note that the 'loop' area of miRNA duplexes is defined as the terminal duplex end that forms upon liberation of the duplex from the hairpin precursor (5' end of 3p strand and 3' end of 5p strand). (C) Comparison of temperature-dependent folding for miRNA hairpins and duplexes vs. normalized miRNA abundance. The average mature miRNA abundance (guide + passenger strand) across development was acquired from a previous study (Panzade et al., 2022) and the $\log_2$ transformed values are presented on the y-axis. We added 0.01 to all miRNA RPM values to allow for $\log_2$ transformation of miRNAs with zero read counts. miRNA intermediates that showed temperature-dependent folding differences are shown as red dots.

Figure S3: Quantification of mismatched or bulged nucleotide frequency for 5p vs. 3p miRNAs based on the hairpin-derived duplex (A-B) or the MFE duplex (C-D) structures, and guide vs. passenger miRNAs based on the hairpin-derived duplex (E-F) or the MFE duplex (G-H) structures. (A-H) Percent of miRNAs containing mismatches (gray) or bulges (purple) at positions (x-axis) relative to the 5' end of the miRNA. 3' terminal nucleotide overhangs were

excluded from this analysis. All *C. elegans* miRNA duplexes (n=190) with annotated guide and passenger strands were included in this analysis.

Figure S4: Predictions of miRNA strand selection. (A) Example let-7 duplexes illustrating nucleotides considered for thermodynamic stability calculations. The terminal two nucleotides (left) or four terminal nucleotides (right) were considered. Ranked-order plots of predicted *C. elegans* miRNA strand asymmetry for (B) MFE duplex structures using unwinding energy, (C) hairpin-derived duplex structures using unwinding energy, (D) End-constrained duplexes using unwinding energy and (E) nearest neighbor energy values. (B-E) Predicted strand ratios were determined using a formula from the twin-drive model [ln(5p/3p) = kΔΔG5p-3p + N5p-N3p], using previously described values for constants (Suzuki et al. 2015) . miRNAs are plotted in order of lowest to highest predicted ln(5p/3p) values. miRNAs above dashed line are predicted 5p dominant and miRNAs below dashed line are predicted 3p dominant. Correct predictions (based on guide strands reported in miRBase) are indicated as black dots, and incorrect predictions are indicated as red dots.

Figure S5: Distribution of ΔΔG values (guide end ΔG – passenger end ΔG) for correct and incorrect predictions of strand selection using different folding methods. ΔΔG values were aquired using the (A) MFE duplex constrained method, (B) hairpin duplex constrained method, (C) duplex ends constrained method or (D) the nearest neighbor method. A positive ΔΔG indicates that the guide end of the duplex was less stable than the passenger end, which is expected to be favorable for miRNA strand selection.

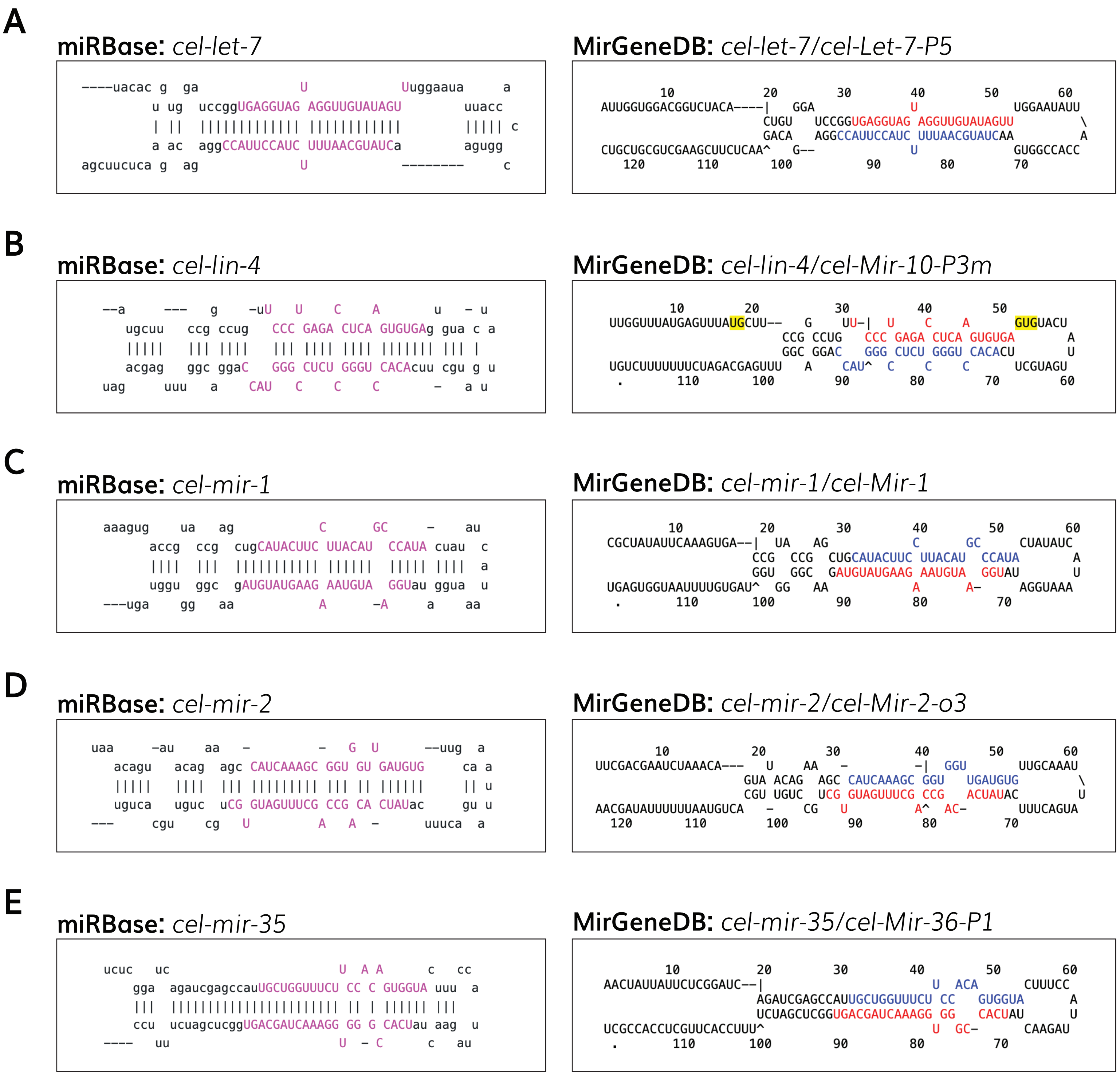
A
miRBase: cel-let-7
MirGeneDB: cel-let-7/cel-Let-7-P5
B
miRBase: cel-lin-4
MirGeneDB: cel-lin-4/cel-Mir-10-P3m
C
miRBase: cel-mir-1
MirGeneDB: cel-mir-1/cel-Mir-1
D
miRBase: cel-mir-2
MirGeneDB: cel-mir-2/cel-Mir-2-o3
E
miRBase: cel-mir-35
MirGeneDB: cel-mir-35/cel-Mir-36-P1

Figure S1

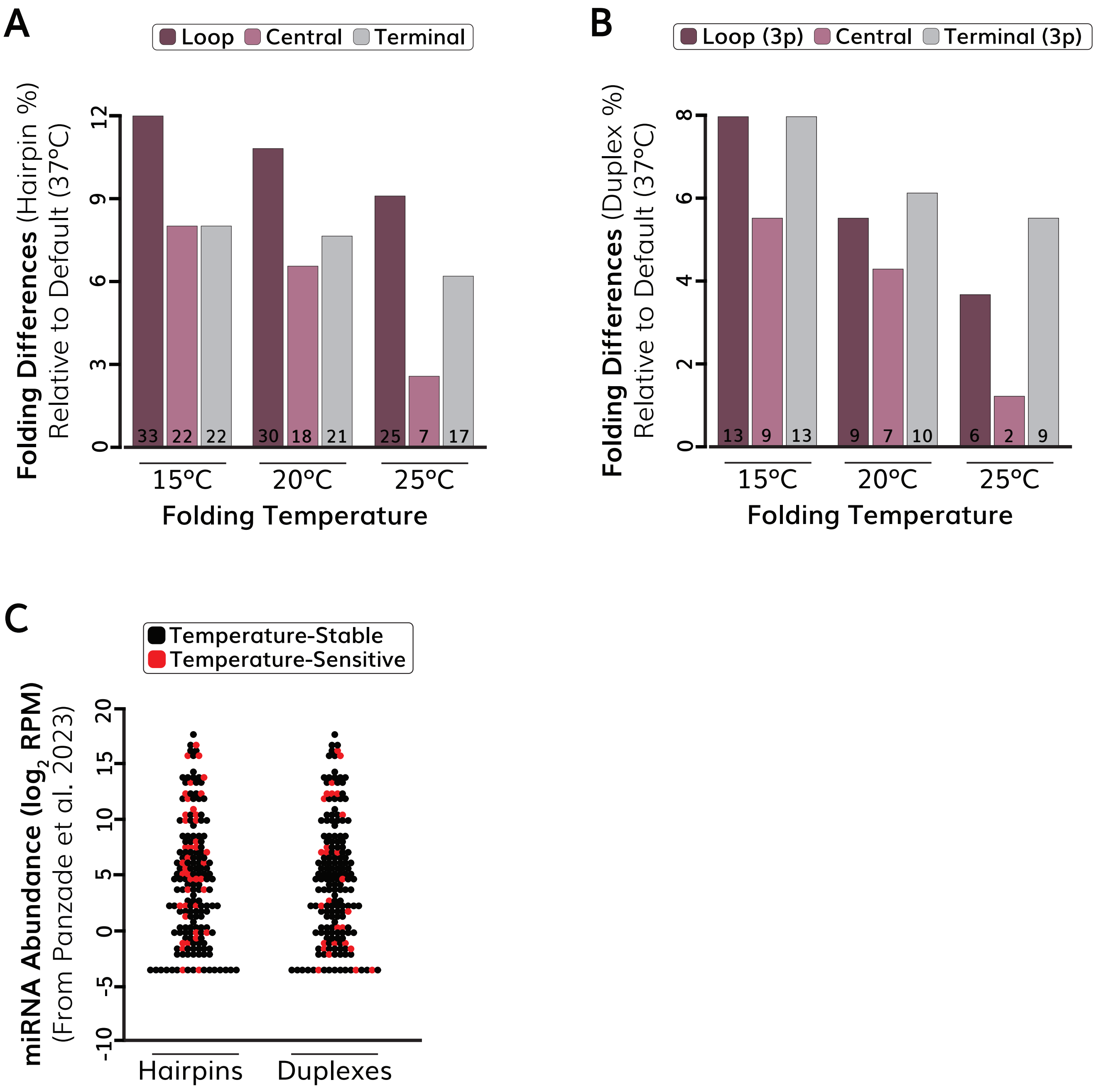
A
Loop
Central
Terminal
Folding Differences (Hairpin %) Relative to Default (37°C)
0
3
6
9
12
33
22
22
30
18
21
25
7
17
15°C
20°C
25°C
Folding Temperature
B
Loop (3p)
Central
Terminal (3p)
Folding Differences (Duplex %) Relative to Default (37°C)
0
2
4
6
8
13
9
13
9
7
10
6
2
9
15°C
20°C
25°C
Folding Temperature
C
Temperature-Stable
Temperature-Sensitive
miRNA Abundance (log2 RPM) (From Panzade et al. 2023)
-10
-5
0
5
10
15
20
Hairpins
Duplexes

Figure S2

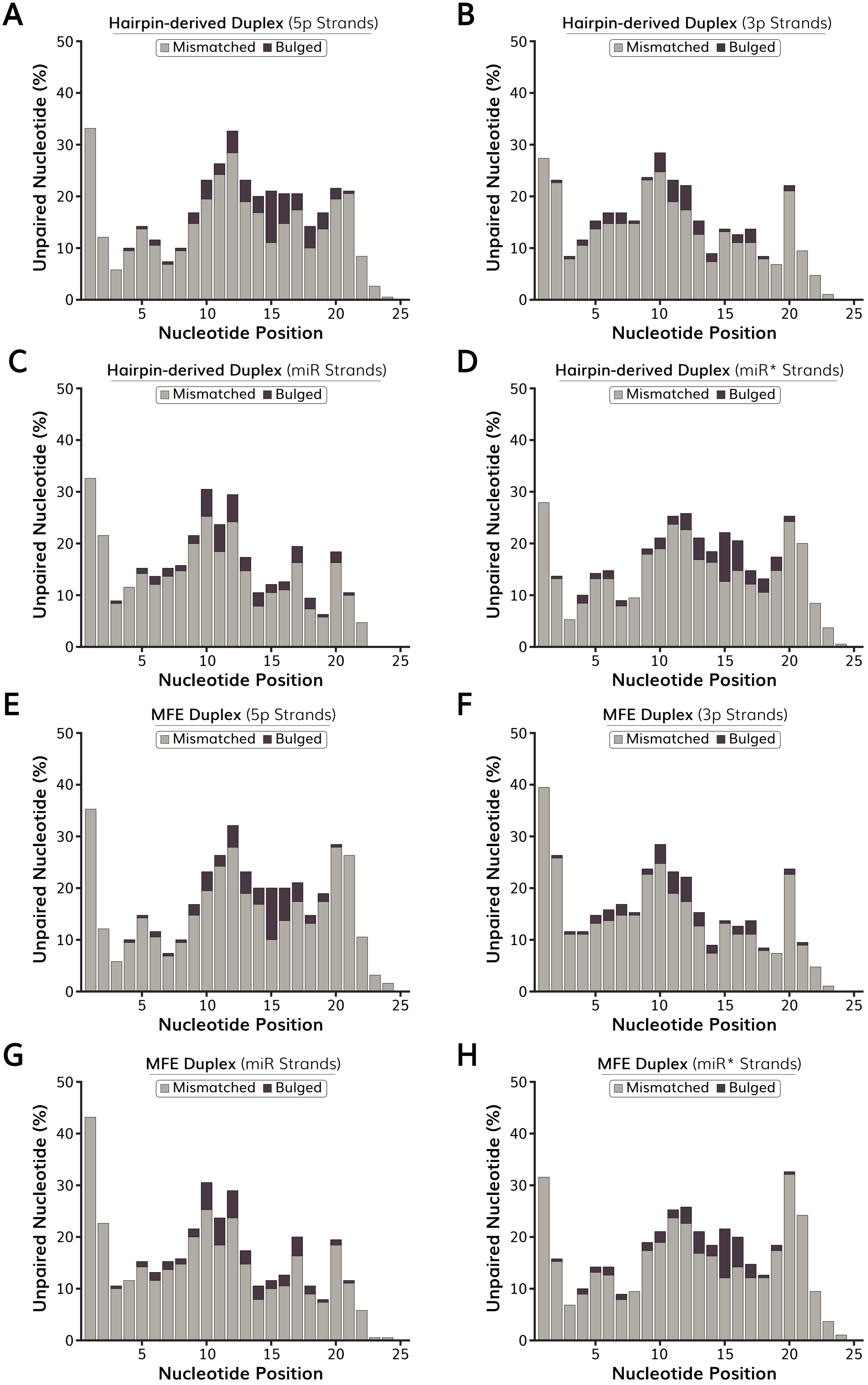
A
Hairpin-derived Duplex (5p Strands)
B
Hairpin-derived Duplex (3p Strands)
C
Hairpin-derived Duplex (miR Strands)
D
Hairpin-derived Duplex (miR* Strands)
E
MFE Duplex (5p Strands)
F
MFE Duplex (3p Strands)
G
MFE Duplex (miR Strands)
H
MFE Duplex (miR* Strands)
Mismatched
Bulged
Unpaired Nucleotide (%)
Nucleotide Position
0
10
20
30
40
50
5
10
15
20
25

Figure S3

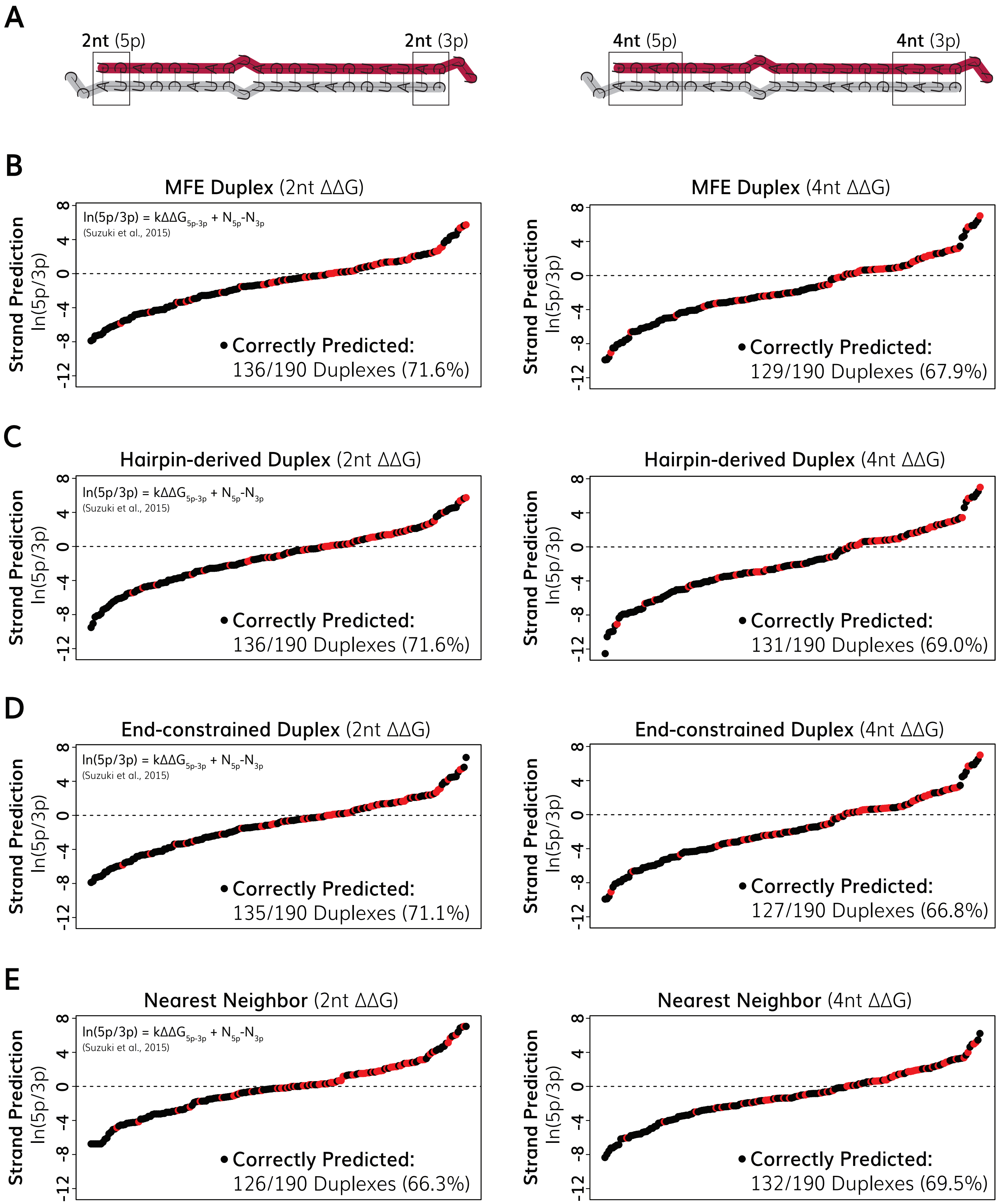
A
2nt (5p)
2nt (3p)
4nt (5p)
4nt (3p)
B
MFE Duplex (2nt ΔΔG)
ln(5p/3p) = kΔΔG5p-3p + N5p-N3p
(Suzuki et al., 2015)
Strand Prediction ln(5p/3p)
Correctly Predicted:
136/190 Duplexes (71.6%)
MFE Duplex (4nt ΔΔG)
Correctly Predicted:
129/190 Duplexes (67.9%)
C
Hairpin-derived Duplex (2nt ΔΔG)
Correctly Predicted:
136/190 Duplexes (71.6%)
Hairpin-derived Duplex (4nt ΔΔG)
Correctly Predicted:
131/190 Duplexes (69.0%)
D
End-constrained Duplex (2nt ΔΔG)
Correctly Predicted:
135/190 Duplexes (71.1%)
End-constrained Duplex (4nt ΔΔG)
Correctly Predicted:
127/190 Duplexes (66.8%)
E
Nearest Neighbor (2nt ΔΔG)
Correctly Predicted:
126/190 Duplexes (66.3%)
Nearest Neighbor (4nt ΔΔG)
Correctly Predicted:
132/190 Duplexes (69.5%)

Figure S4

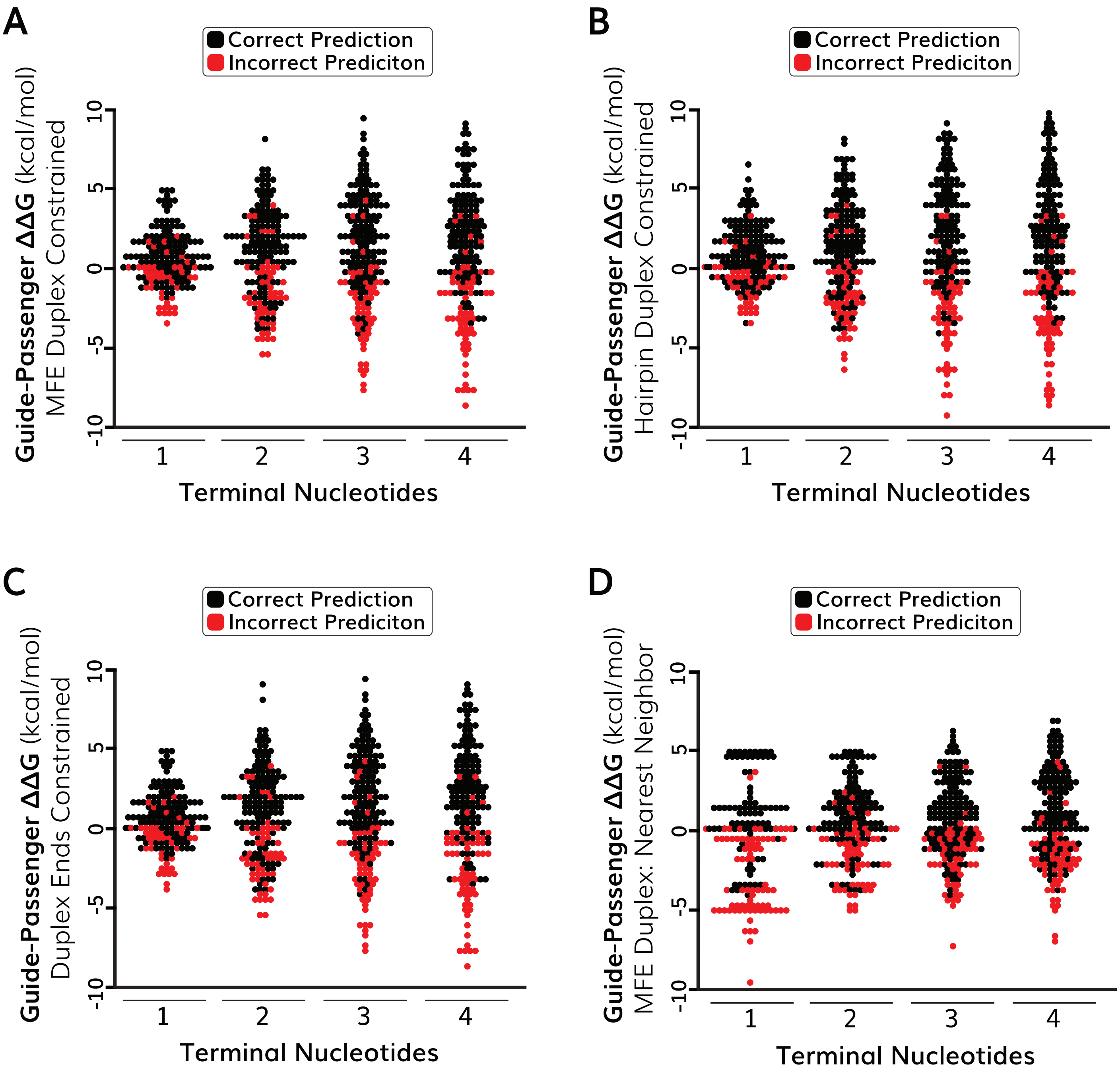
A
Correct Prediction
Incorrect Prediciton
Guide-Passenger ΔΔG (kcal/mol)
MFE Duplex Constrained
10
5
0
-5
-10
1
2
3
4
Terminal Nucleotides
B
Correct Prediction
Incorrect Prediciton
Guide-Passenger ΔΔG (kcal/mol)
Hairpin Duplex Constrained
10
5
0
-5
-10
1
2
3
4
Terminal Nucleotides
C
Correct Prediction
Incorrect Prediciton
Guide-Passenger ΔΔG (kcal/mol)
Duplex Ends Constrained
10
5
0
-5
-10
1
2
3
4
Terminal Nucleotides
D
Correct Prediction
Incorrect Prediciton
Guide-Passenger ΔΔG (kcal/mol)
MFE Duplex: Nearest Neighbor
10
5
0
-5
-10
1
2
3
4
Terminal Nucleotides

Figure S5

Table S1: *C. elegans* miRNA hairpins folded at different temperatures.

miRNA	Hairpin (15°C)	Hairpin (20°C)	Hairpin (25°C)	Hairpin (37°C)
let-7	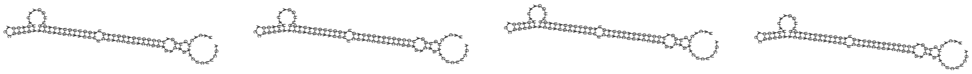			
lin-4	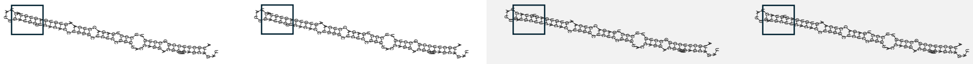			
lsy-6	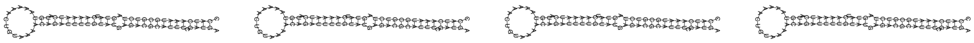			
mir-1018	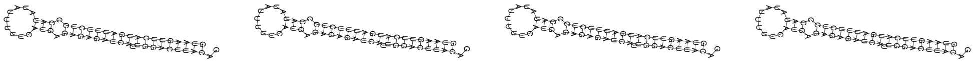			
mir-1019	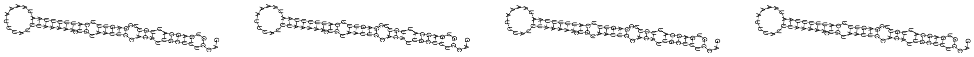			
mir-1020	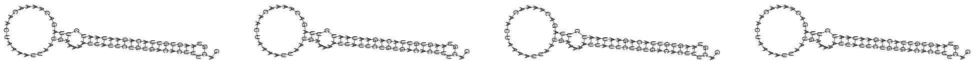			
mir-1021	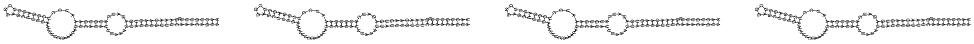			
mir-1022	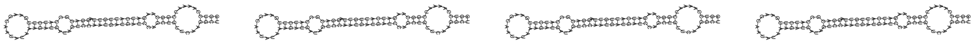			
mir-12134	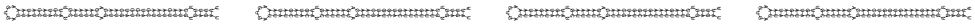			
mir-124	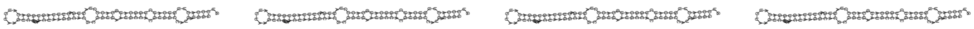			
mir-1817	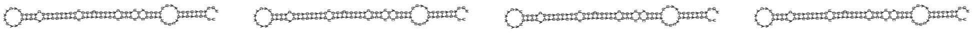			
mir-1818	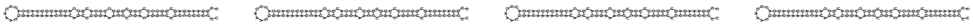			
mir-1819	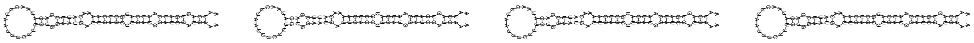			
mir-1820	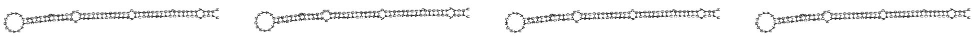			
mir-1821	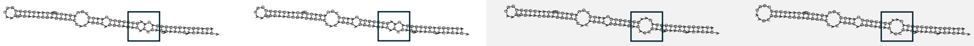			
mir-1822	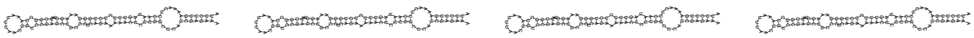			
mir-1823	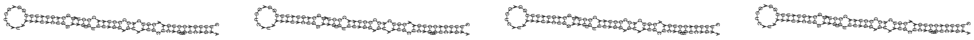			
mir-1824	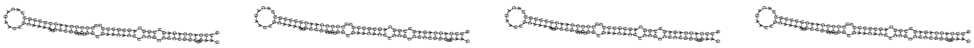			

mir-1828	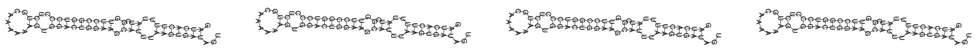
mir-1829a	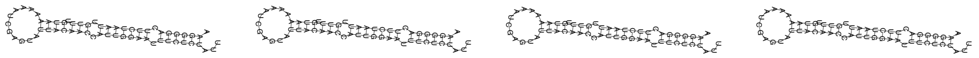
mir-1829b	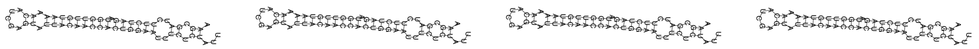
mir-1829c	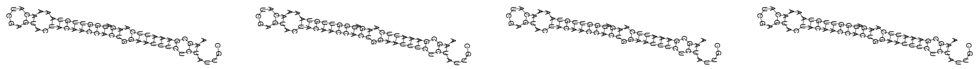
mir-1830	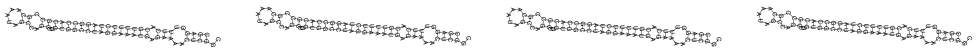
mir-1832a	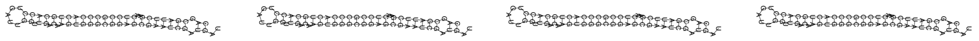
mir-1832b	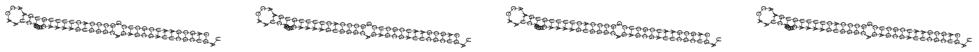
mir-1833	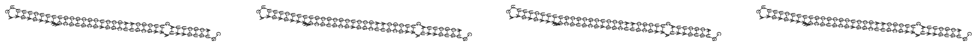
mir-1	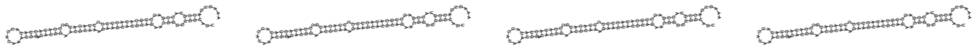
mir-2207	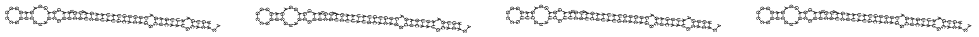
mir-2208a	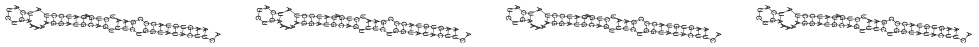
mir-2208b	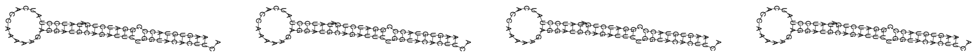
mir-2209a	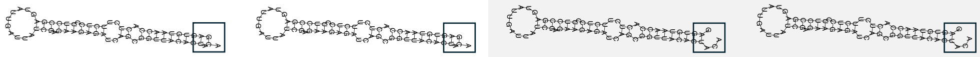
mir-2209b	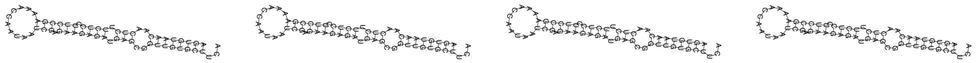
mir-2209c	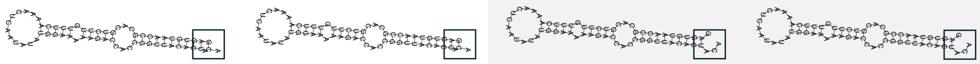
mir-2210	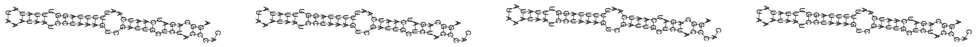
mir-2211	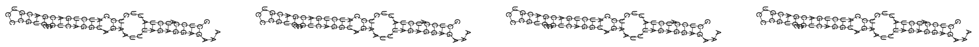
mir-2212	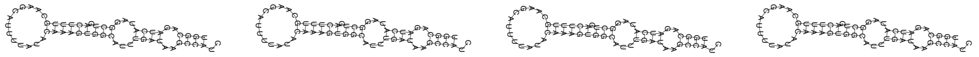
mir-2213	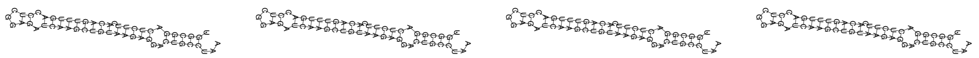

mir-2215
mir-2216
mir-2217a
mir-2217b-1
mir-2217b-2
mir-2217b-3
mir-2217b-4
mir-2218a
mir-2218b
mir-2219
mir-2220
mir-2221
mir-228
mir-229
mir-230
mir-231
mir-232
mir-233
mir-234

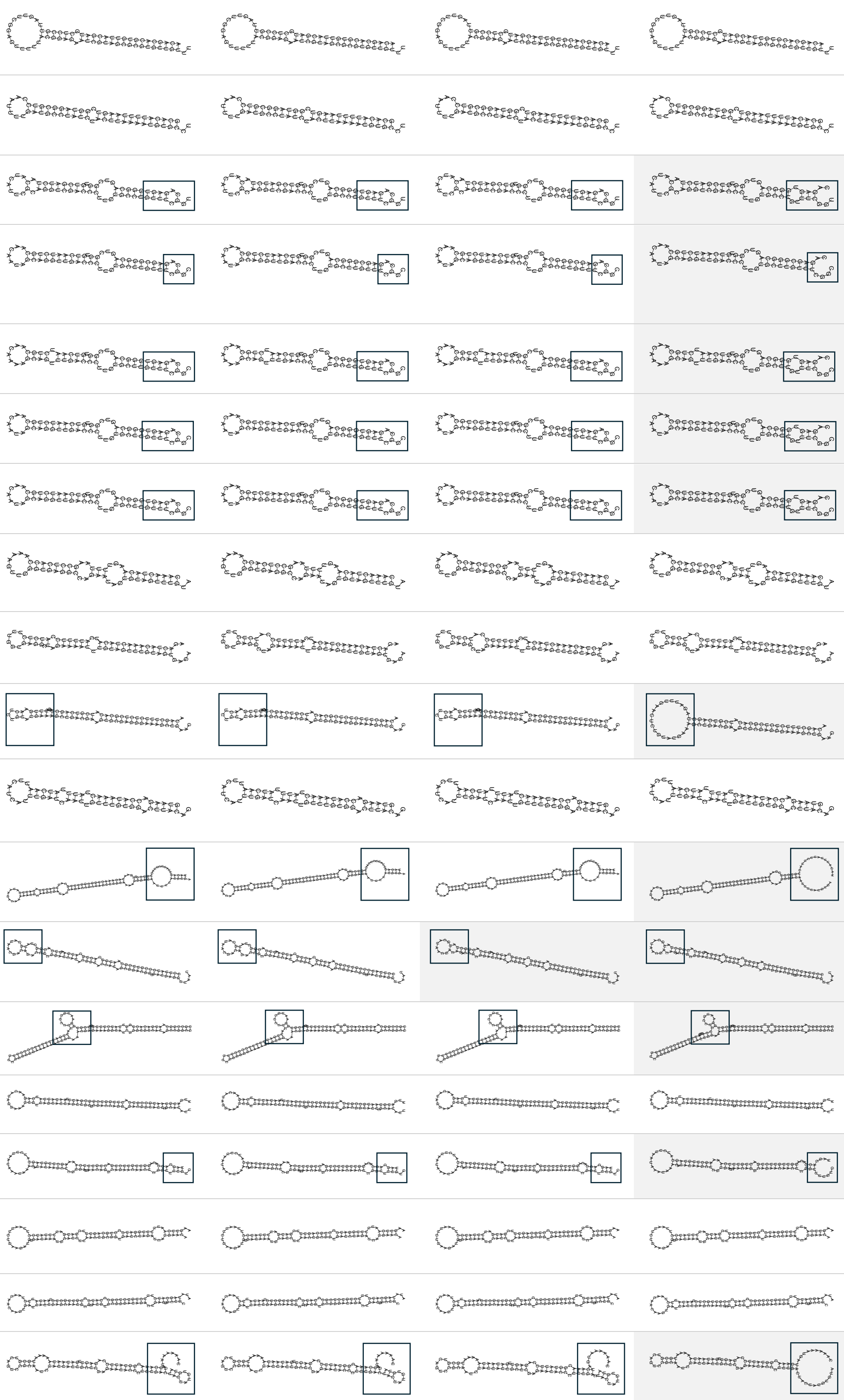

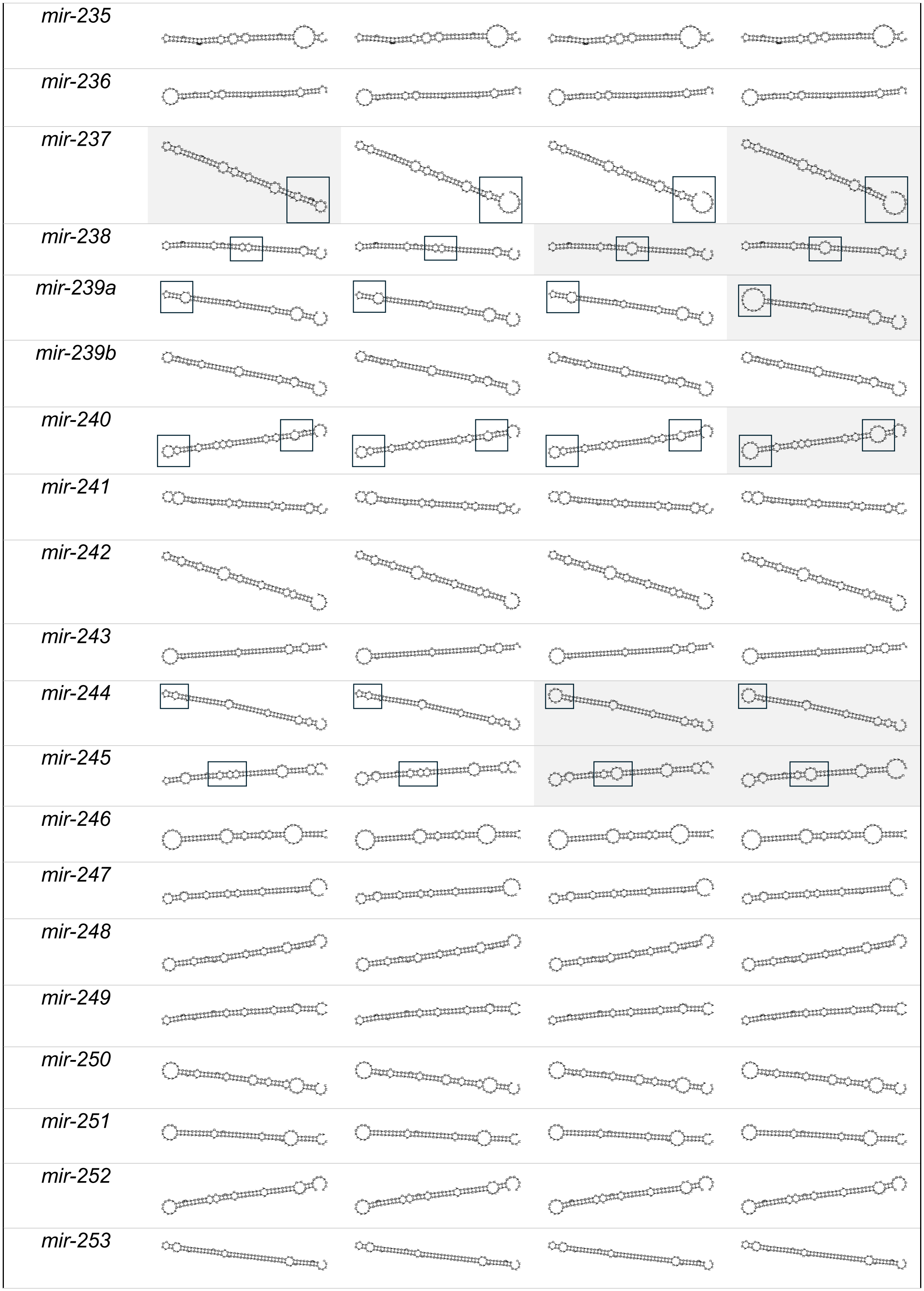
mir-235
mir-236
mir-237
mir-238
mir-239a
mir-239b
mir-240
mir-241
mir-242
mir-243
mir-244
mir-245
mir-246
mir-247
mir-248
mir-249
mir-250
mir-251
mir-252
mir-253

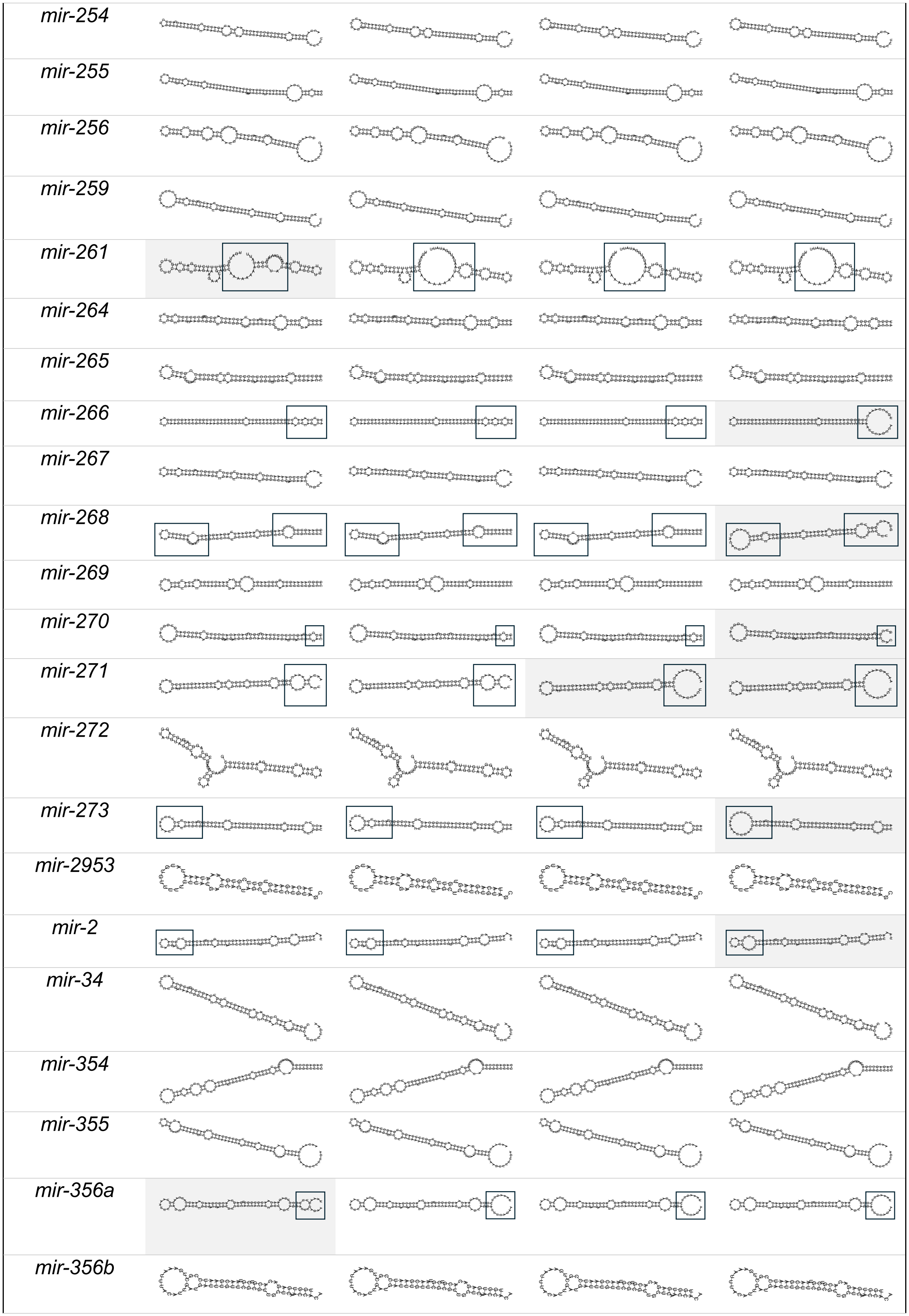
mir-254
mir-255
mir-256
mir-259
mir-261
mir-264
mir-265
mir-266
mir-267
mir-268
mir-269
mir-270
mir-271
mir-272
mir-273
mir-2953
mir-2
mir-34
mir-354
mir-355
mir-356a
mir-356b

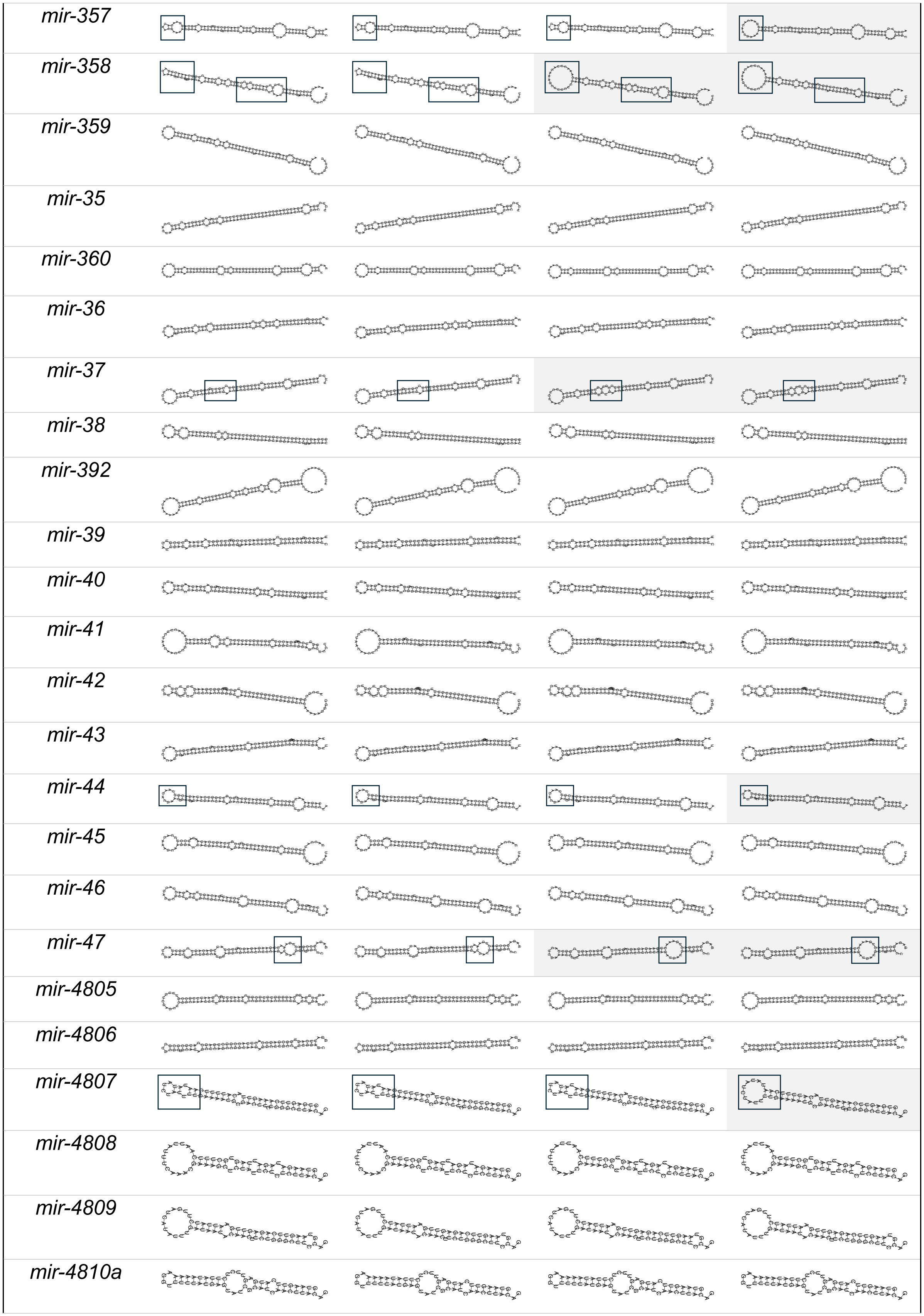
mir-357
mir-358
mir-359
mir-35
mir-360
mir-36
mir-37
mir-38
mir-392
mir-39
mir-40
mir-41
mir-42
mir-43
mir-44
mir-45
mir-46
mir-47
mir-4805
mir-4806
mir-4807
mir-4808
mir-4809
mir-4810a

mir-4810b
mir-4811
mir-4812
mir-4813
mir-4814
mir-4815
mir-4816
mir-48
mir-4920
mir-4921
mir-4922-1
mir-4922-2
mir-4923a
mir-4923b
mir-4924
mir-4925
mir-4926
mir-4927
mir-4929
mir-4930
mir-4931

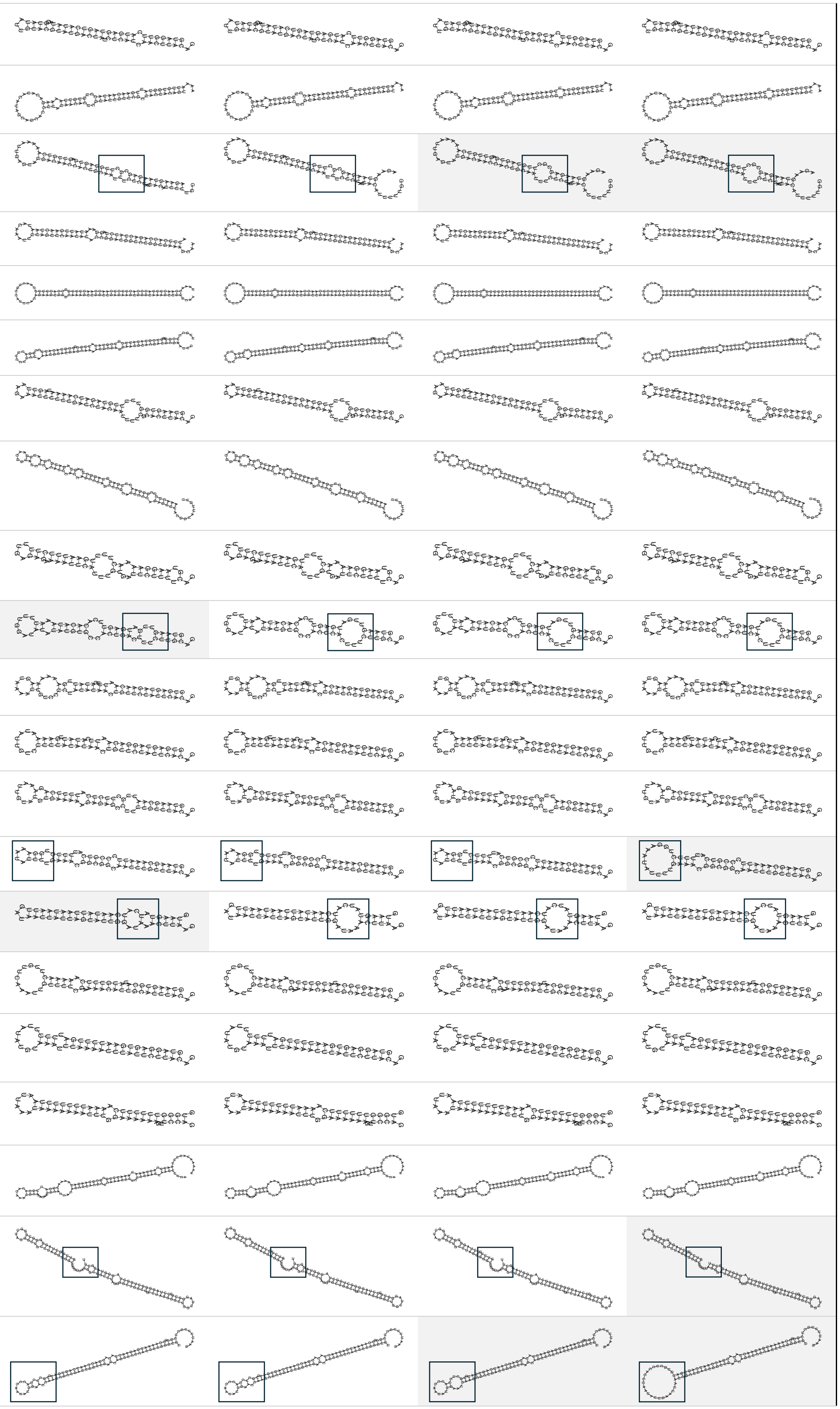

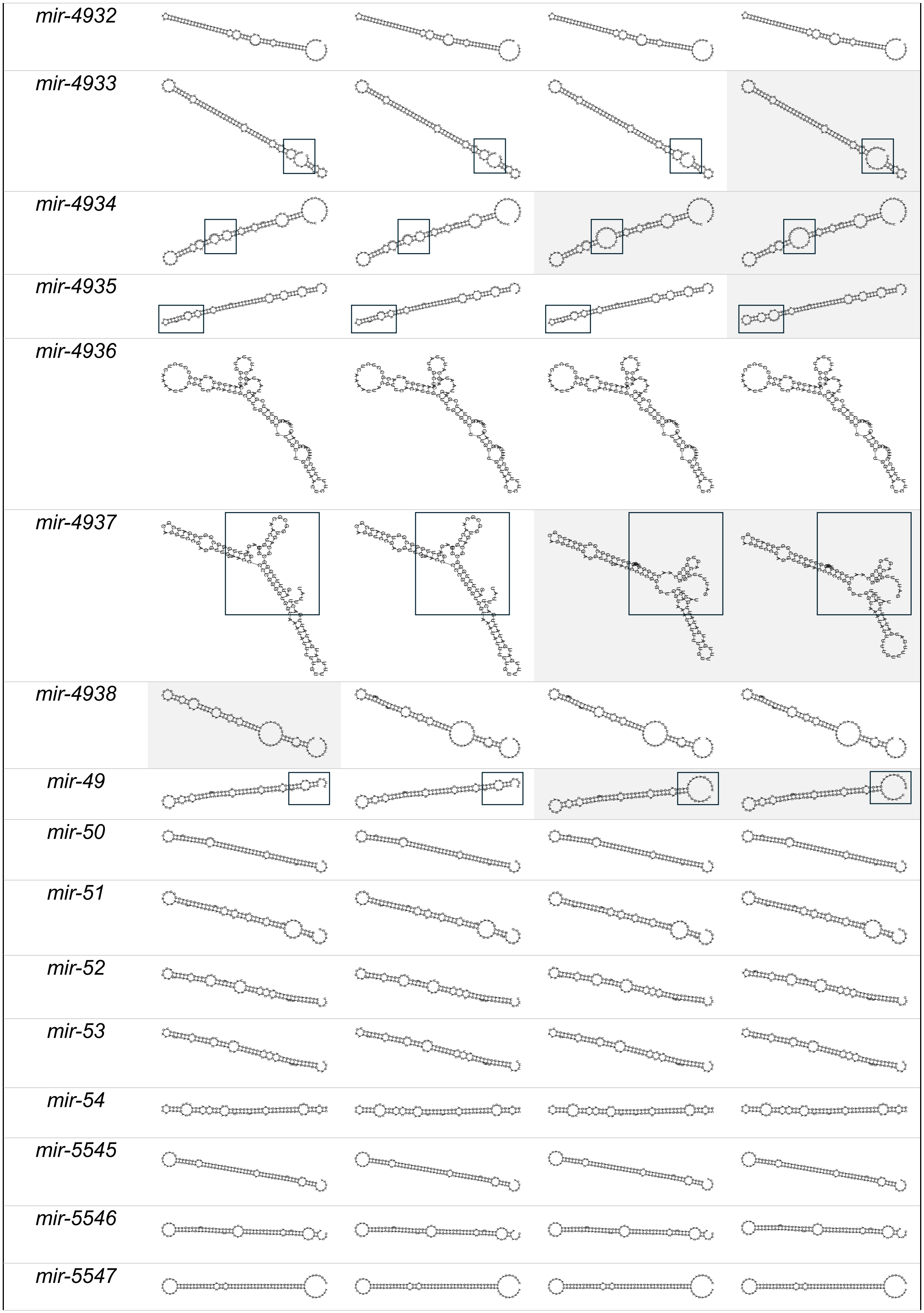
mir-4932
mir-4933
mir-4934
mir-4935
mir-4936
mir-4937
mir-4938
mir-49
mir-50
mir-51
mir-52
mir-53
mir-54
mir-5545
mir-5546
mir-5547

mir-5548	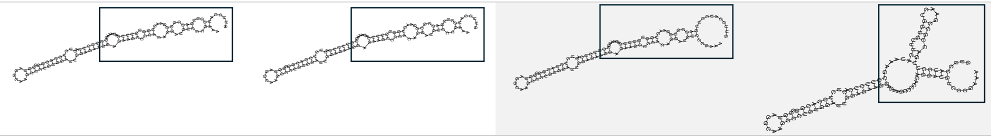
mir-5549	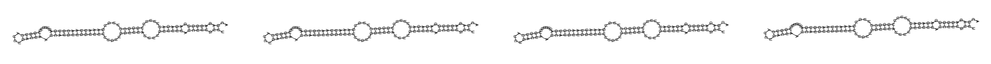
mir-5550	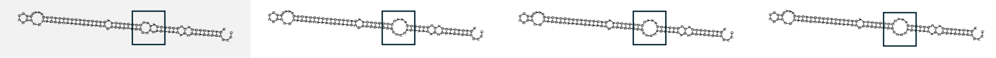
mir-5551	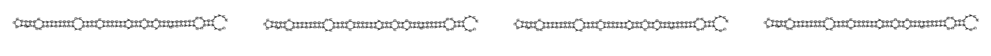
mir-5552	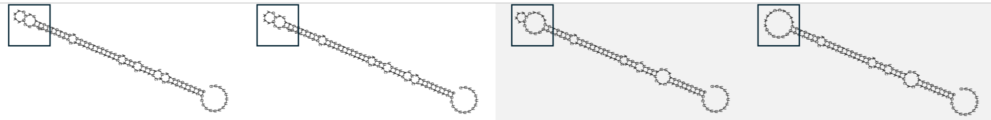
mir-5553	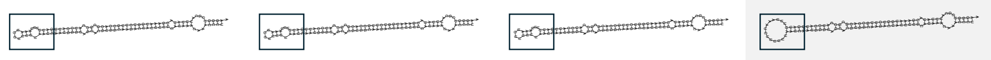
mir-5592-1	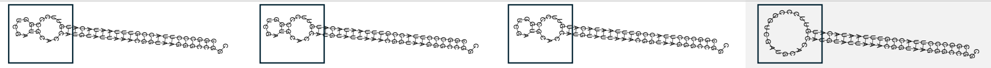
mir-5592-2	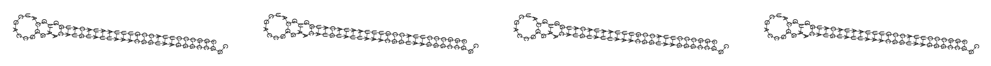
mir-5593-1	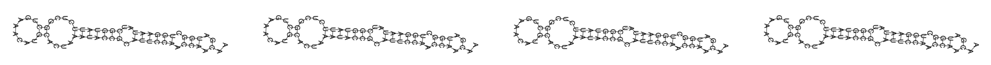
mir-5593-2	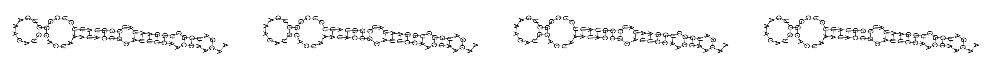
mir-5594	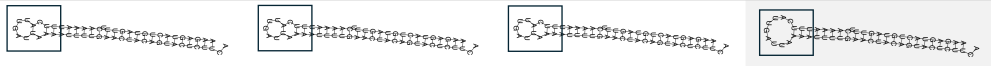
mir-5595	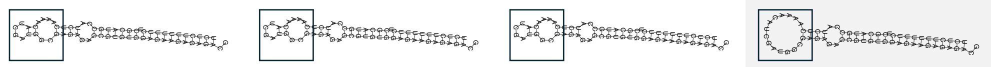
mir-55	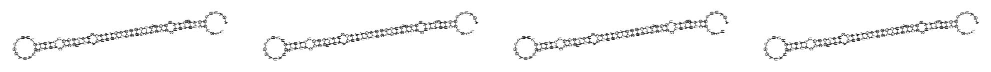
mir-55b	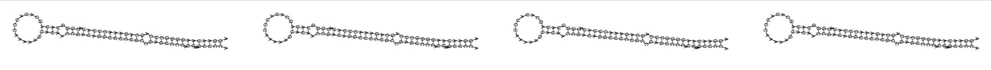
mir-56	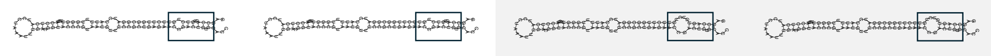
mir-57	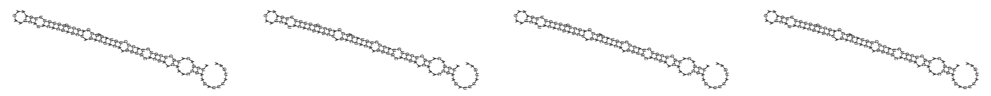
mir-58a	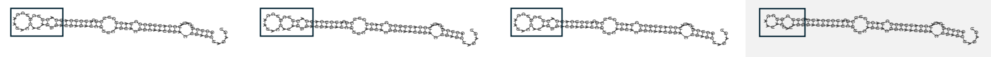
mir-58b	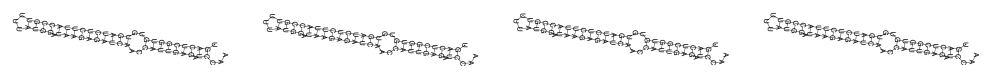
mir-58c	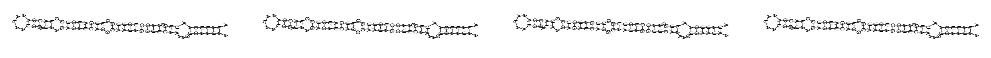
mir-59	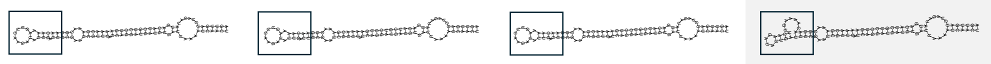
mir-60	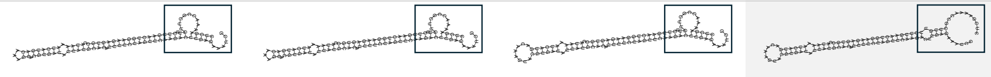
mir-61	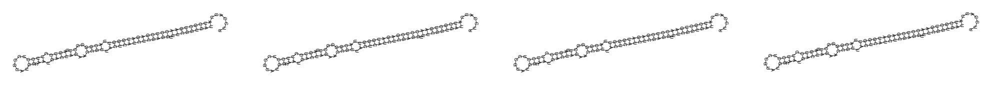

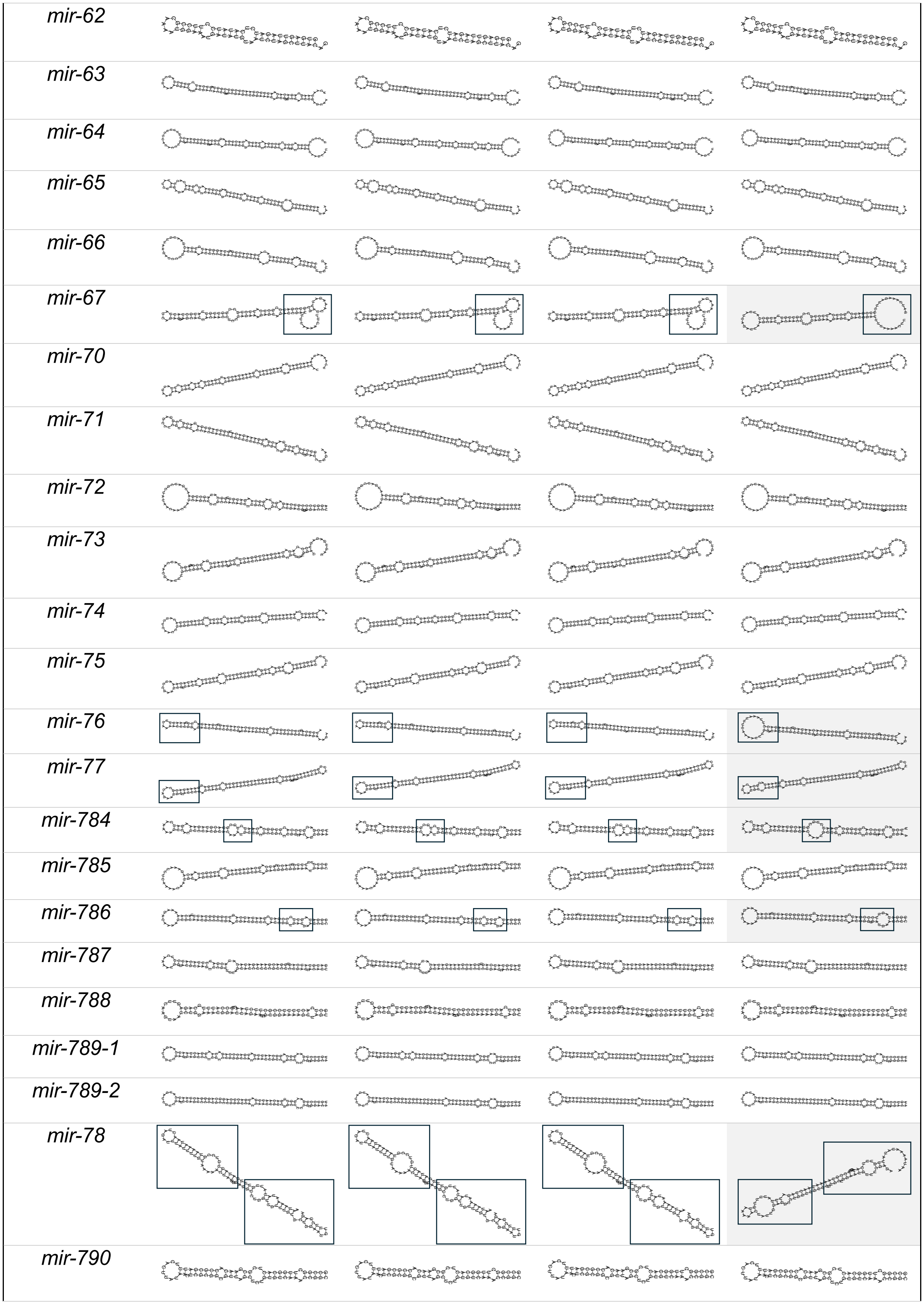
mir-62
mir-63
mir-64
mir-65
mir-66
mir-67
mir-70
mir-71
mir-72
mir-73
mir-74
mir-75
mir-76
mir-77
mir-784
mir-785
mir-786
mir-787
mir-788
mir-789-1
mir-789-2
mir-78
mir-790

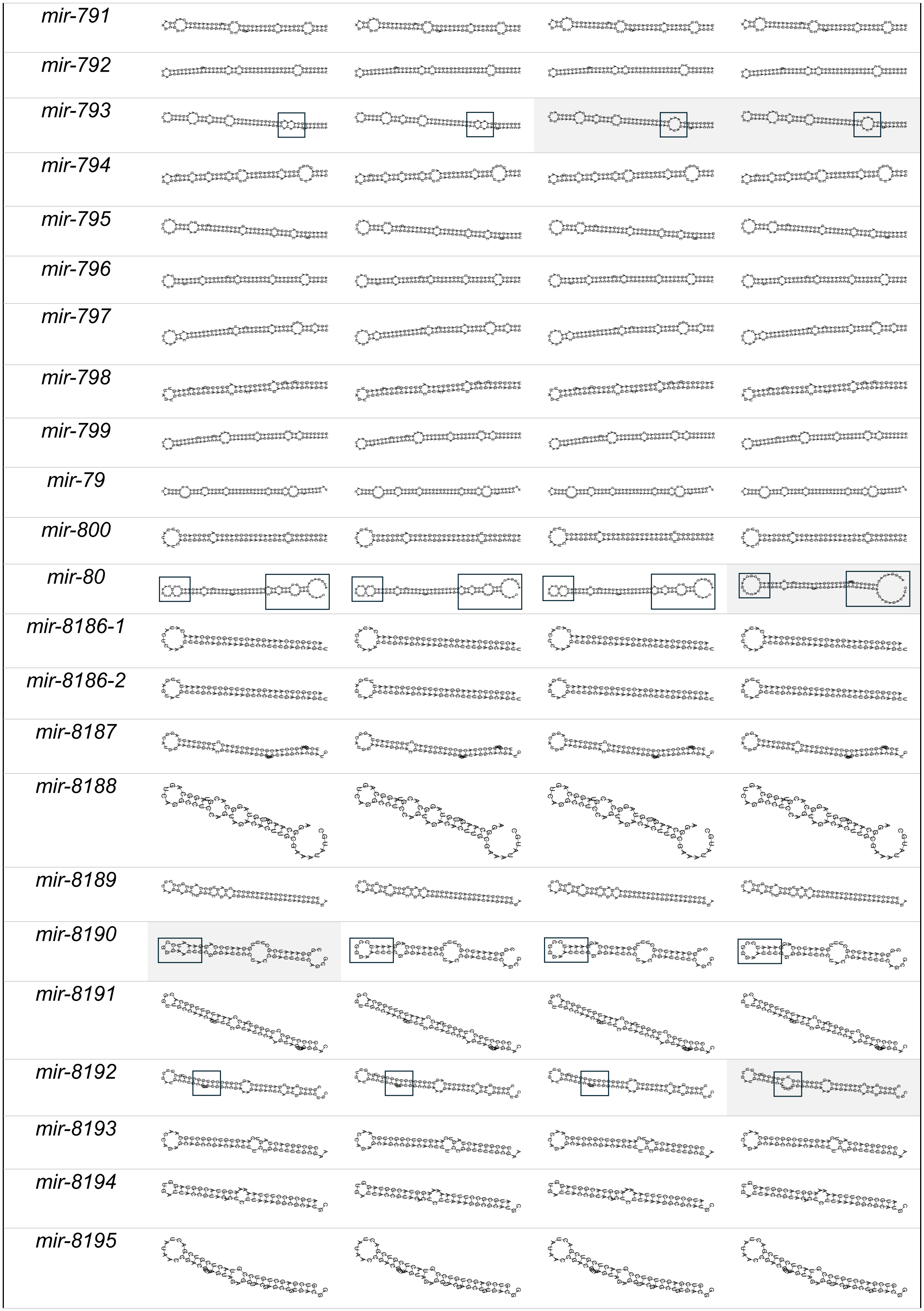
mir-791
mir-792
mir-793
mir-794
mir-795
mir-796
mir-797
mir-798
mir-799
mir-79
mir-800
mir-80
mir-8186-1
mir-8186-2
mir-8187
mir-8188
mir-8189
mir-8190
mir-8191
mir-8192
mir-8193
mir-8194
mir-8195

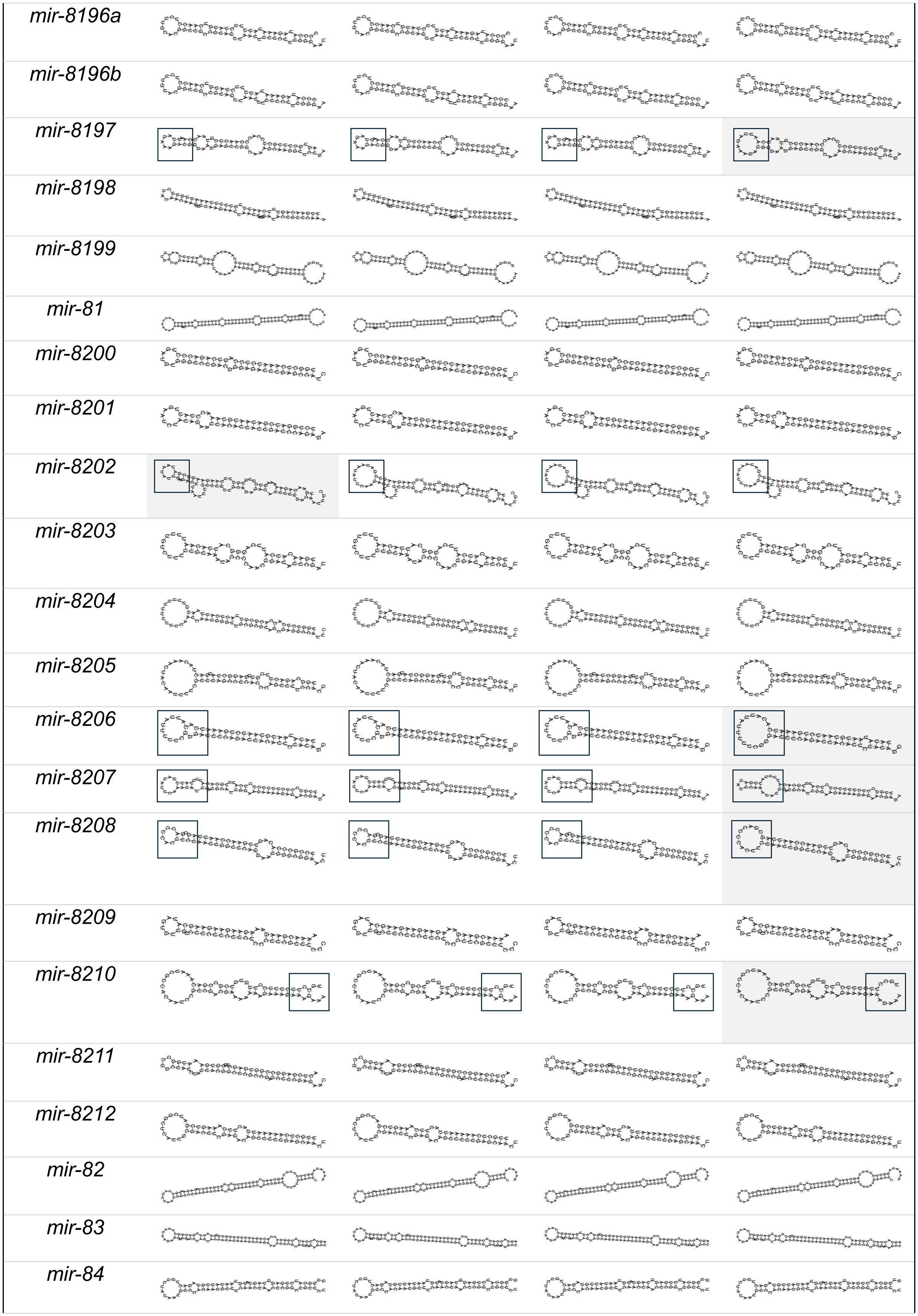
mir-8196a
mir-8196b
mir-8197
mir-8198
mir-8199
mir-81
mir-8200
mir-8201
mir-8202
mir-8203
mir-8204
mir-8205
mir-8206
mir-8207
mir-8208
mir-8209
mir-8210
mir-8211
mir-8212
mir-82
mir-83
mir-84

mir-85	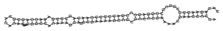	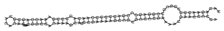	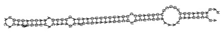	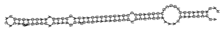
mir-86	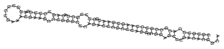	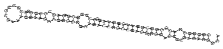	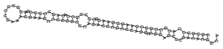	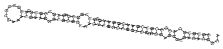
mir-87	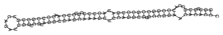	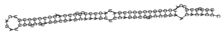	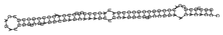	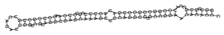
mir-90	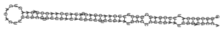	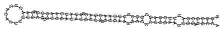	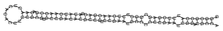	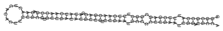

Table S2: *C. elegans* miRNA duplexes folded at different temperatures

miRNA	Duplex (15°C)	Duplex (20°C)	Duplex (25°C)	Duplex (37°C)
let-7	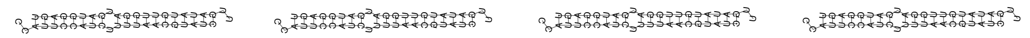			
lin-4	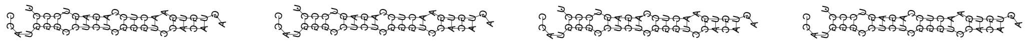			
lsy-6	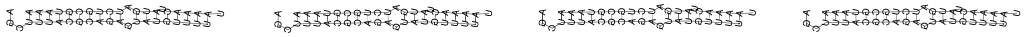			
mir-1019	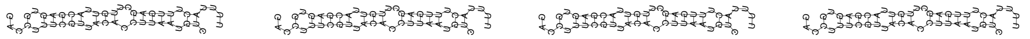			
mir-1020	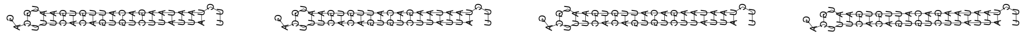			
mir-1022	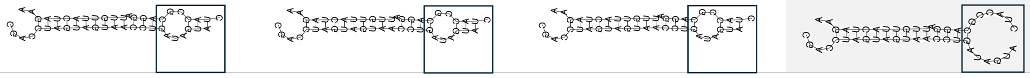			
mir-124	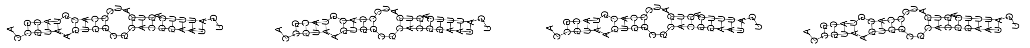			
mir-1819	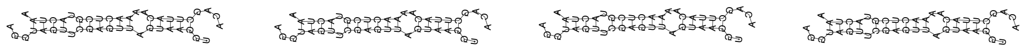			
mir-1820	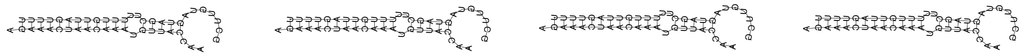			
mir-1821	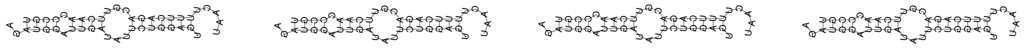			
mir-1822	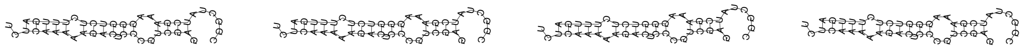			
mir-1823	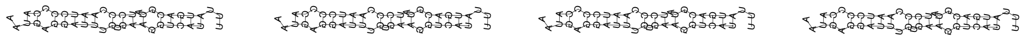			
mir-1824	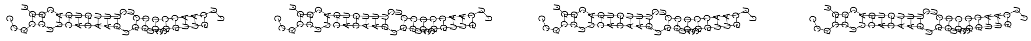			
mir-1829a	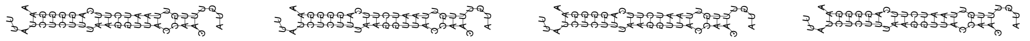			
mir-1829b	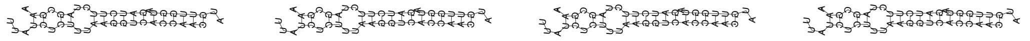			
mir-1829c	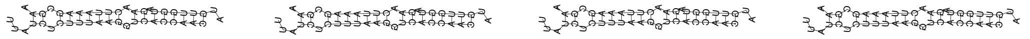			
mir-1830	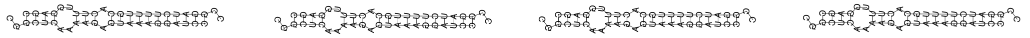			
mir-1832a	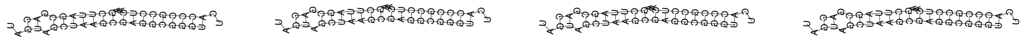			
mir-1832b	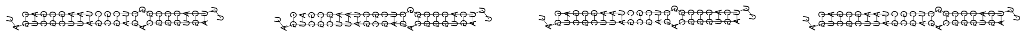			
mir-1	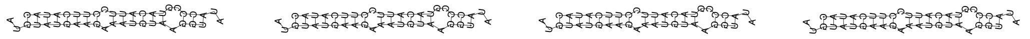			
mir-2207	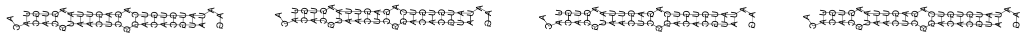			
mir-2208a	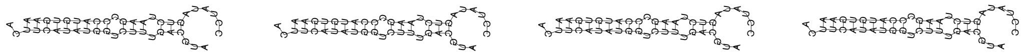			
mir-2208b	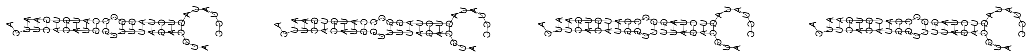			

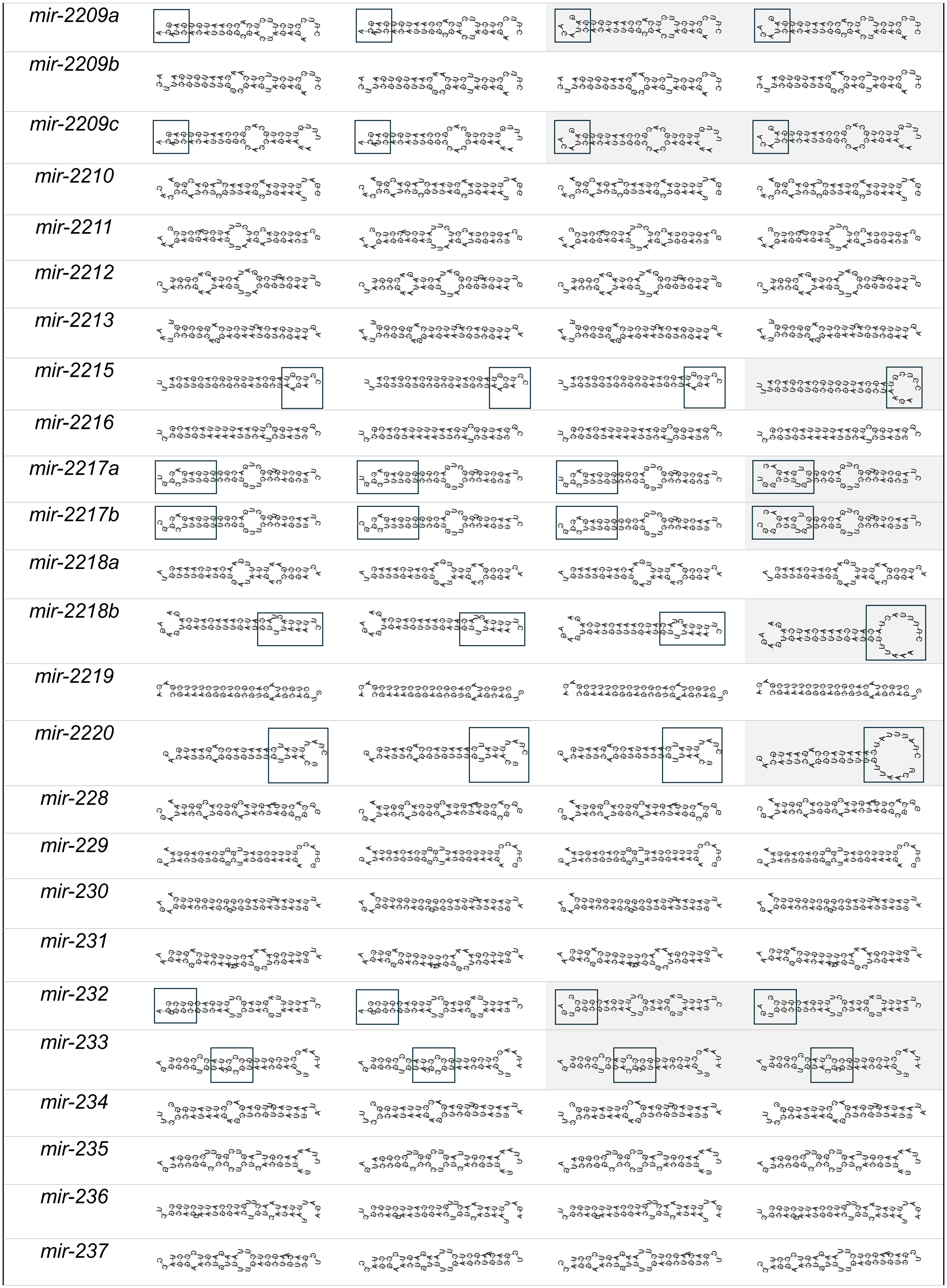
mir-2209a
mir-2209b
mir-2209c
mir-2210
mir-2211
mir-2212
mir-2213
mir-2215
mir-2216
mir-2217a
mir-2217b
mir-2218a
mir-2218b
mir-2219
mir-2220
mir-228
mir-229
mir-230
mir-231
mir-232
mir-233
mir-234
mir-235
mir-236
mir-237

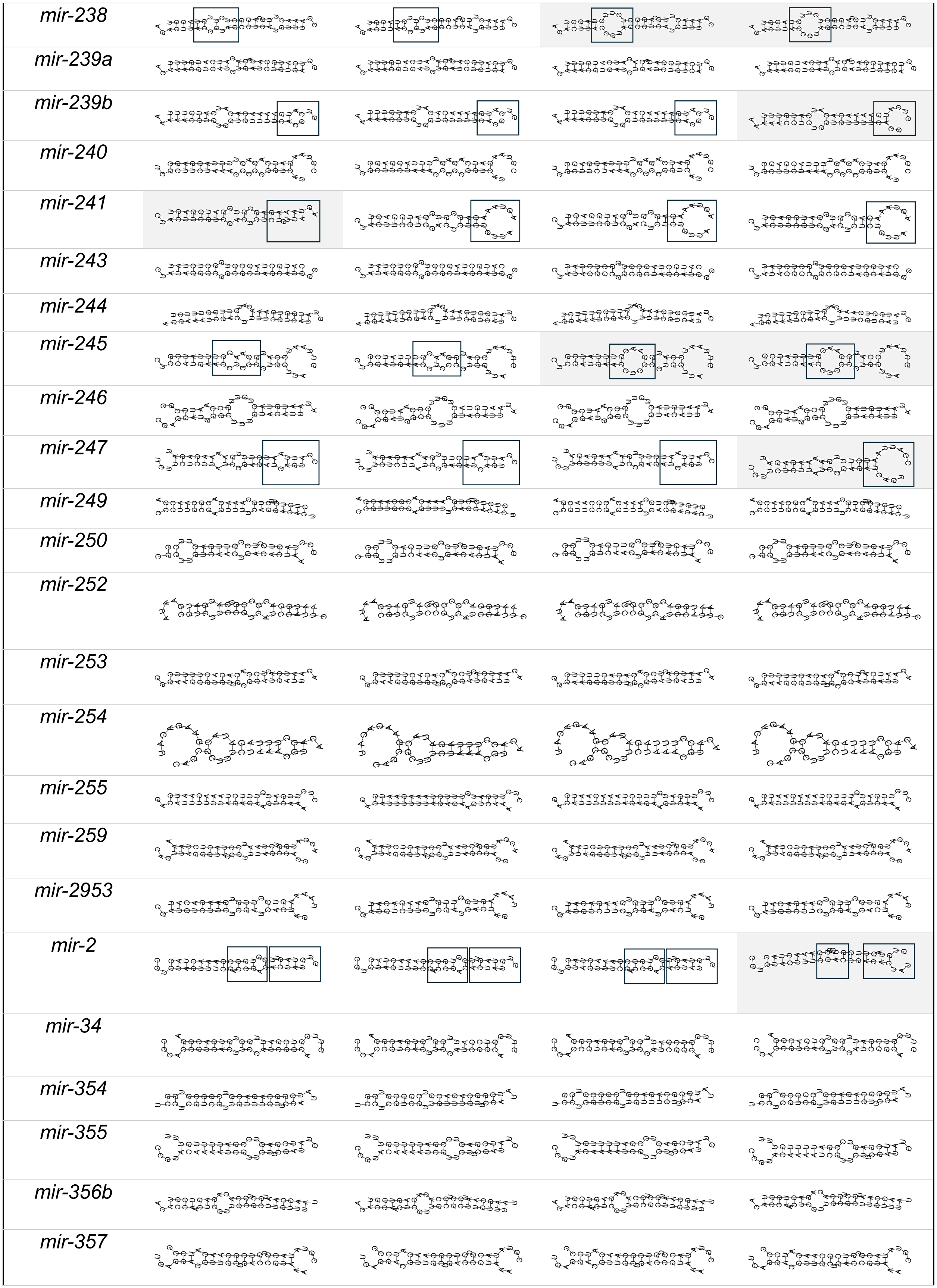
mir-238
mir-239a
mir-239b
mir-240
mir-241
mir-243
mir-244
mir-245
mir-246
mir-247
mir-249
mir-250
mir-252
mir-253
mir-254
mir-255
mir-259
mir-2953
mir-2
mir-34
mir-354
mir-355
mir-356b
mir-357

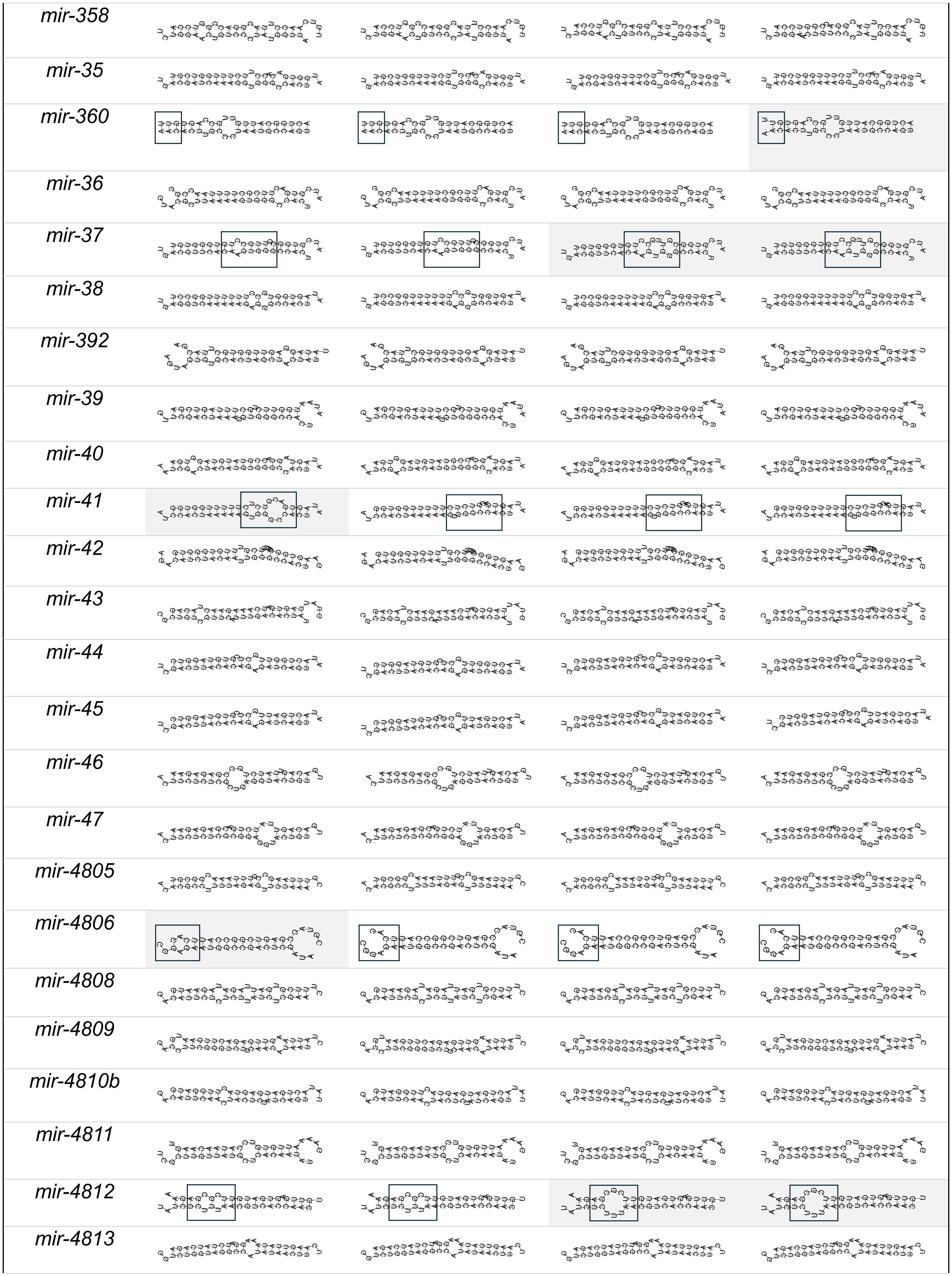
mir-358
mir-35
mir-360
mir-36
mir-37
mir-38
mir-392
mir-39
mir-40
mir-41
mir-42
mir-43
mir-44
mir-45
mir-46
mir-47
mir-4805
mir-4806
mir-4808
mir-4809
mir-4810b
mir-4811
mir-4812
mir-4813

mir-4814	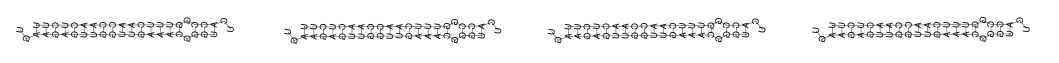
mir-4816	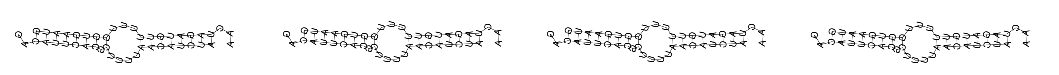
mir-48	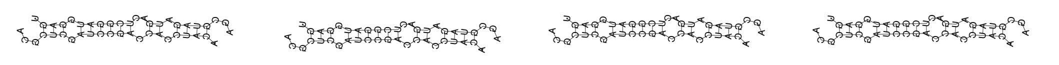
mir-49	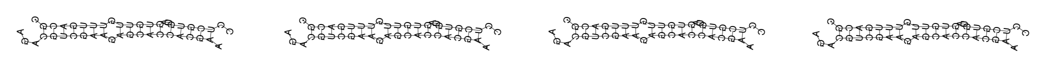
mir-50	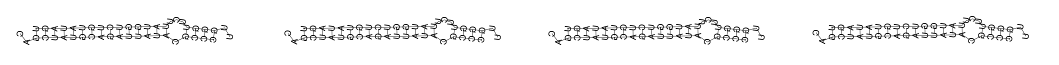
mir-51	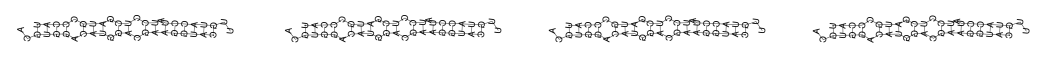
mir-52	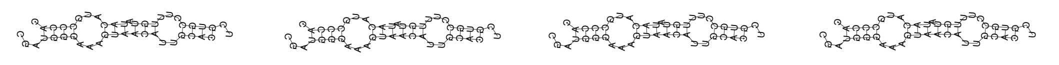
mir-53	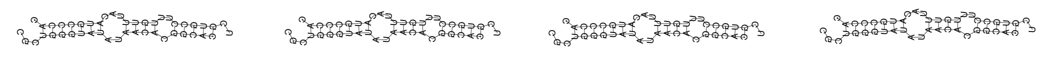
mir-54	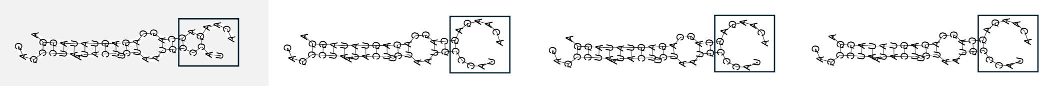
mir-5545	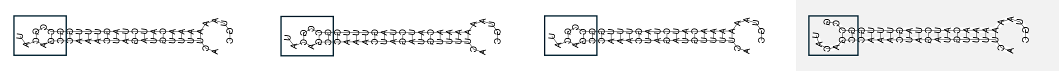
mir-5546	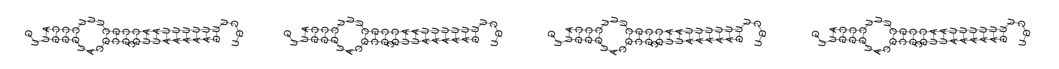
mir-5547	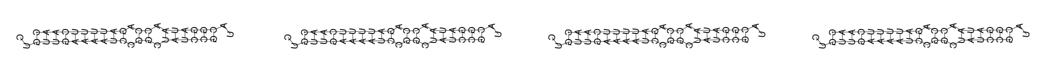
mir-5548	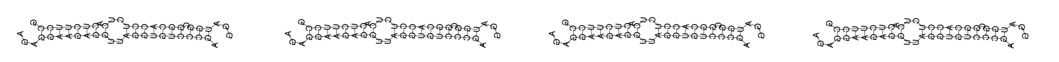
mir-5549	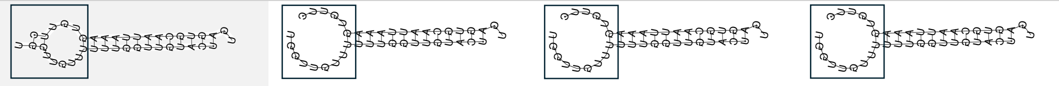
mir-5550	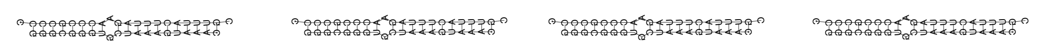
mir-5551	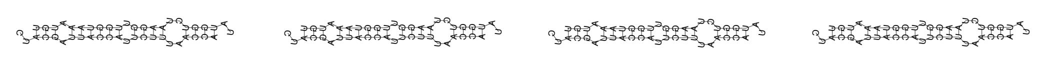
mir-5552	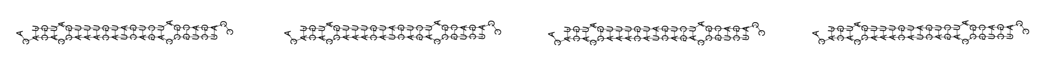
mir-5553	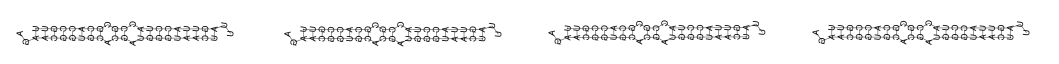
mir-5592	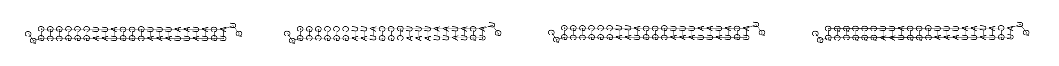
mir-5593	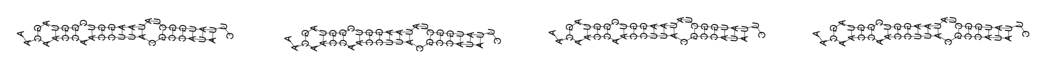
mir-5594	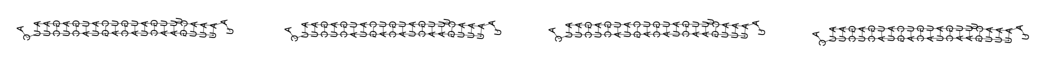
mir-5595	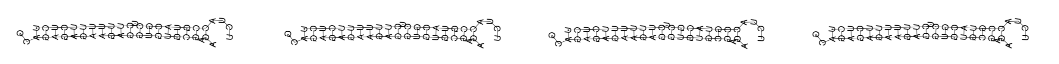
mir-55	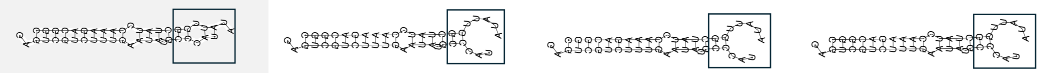
mir-56	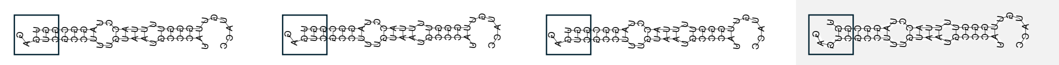

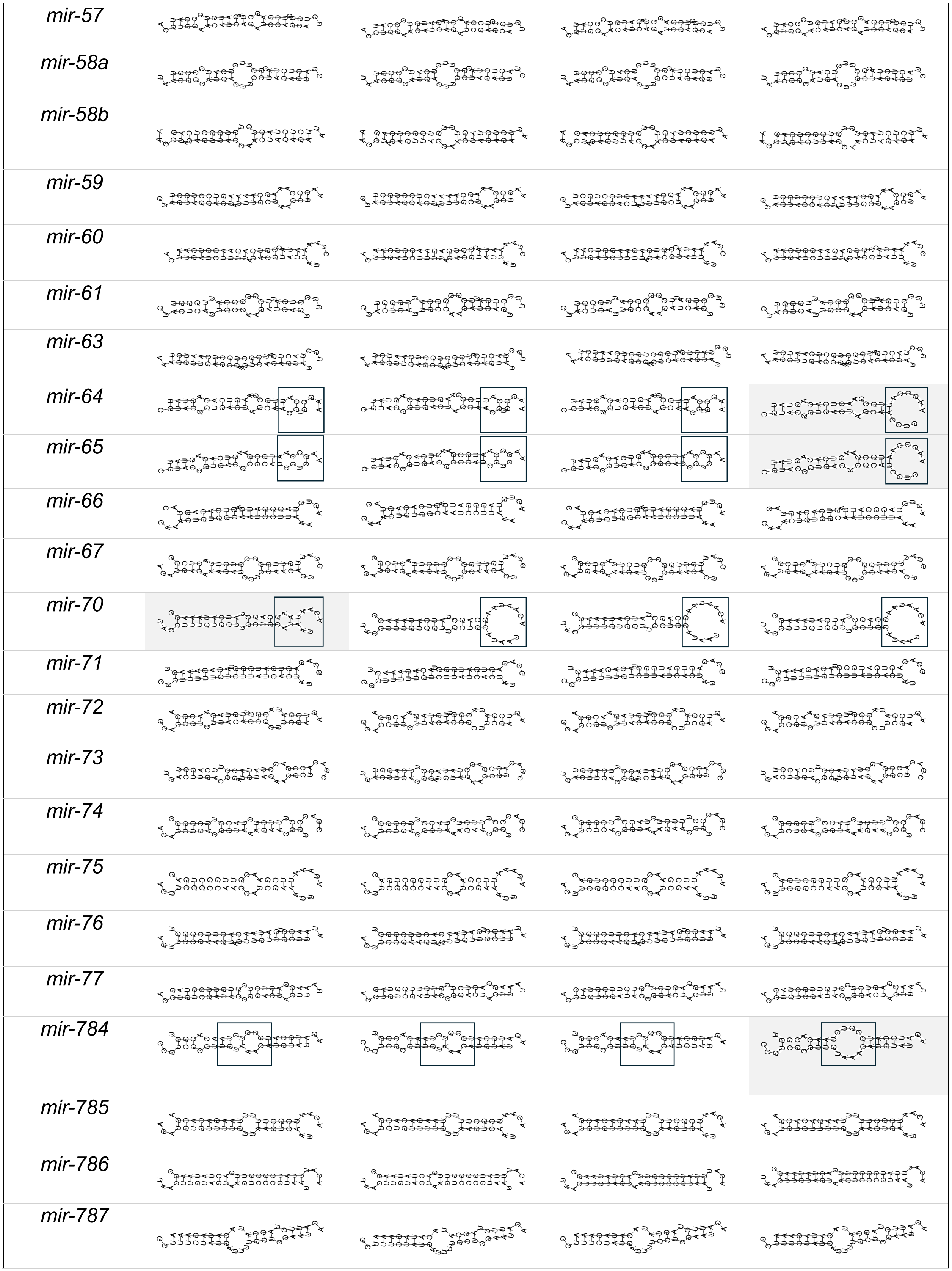
mir-57
mir-58a
mir-58b
mir-59
mir-60
mir-61
mir-63
mir-64
mir-65
mir-66
mir-67
mir-70
mir-71
mir-72
mir-73
mir-74
mir-75
mir-76
mir-77
mir-784
mir-785
mir-786
mir-787

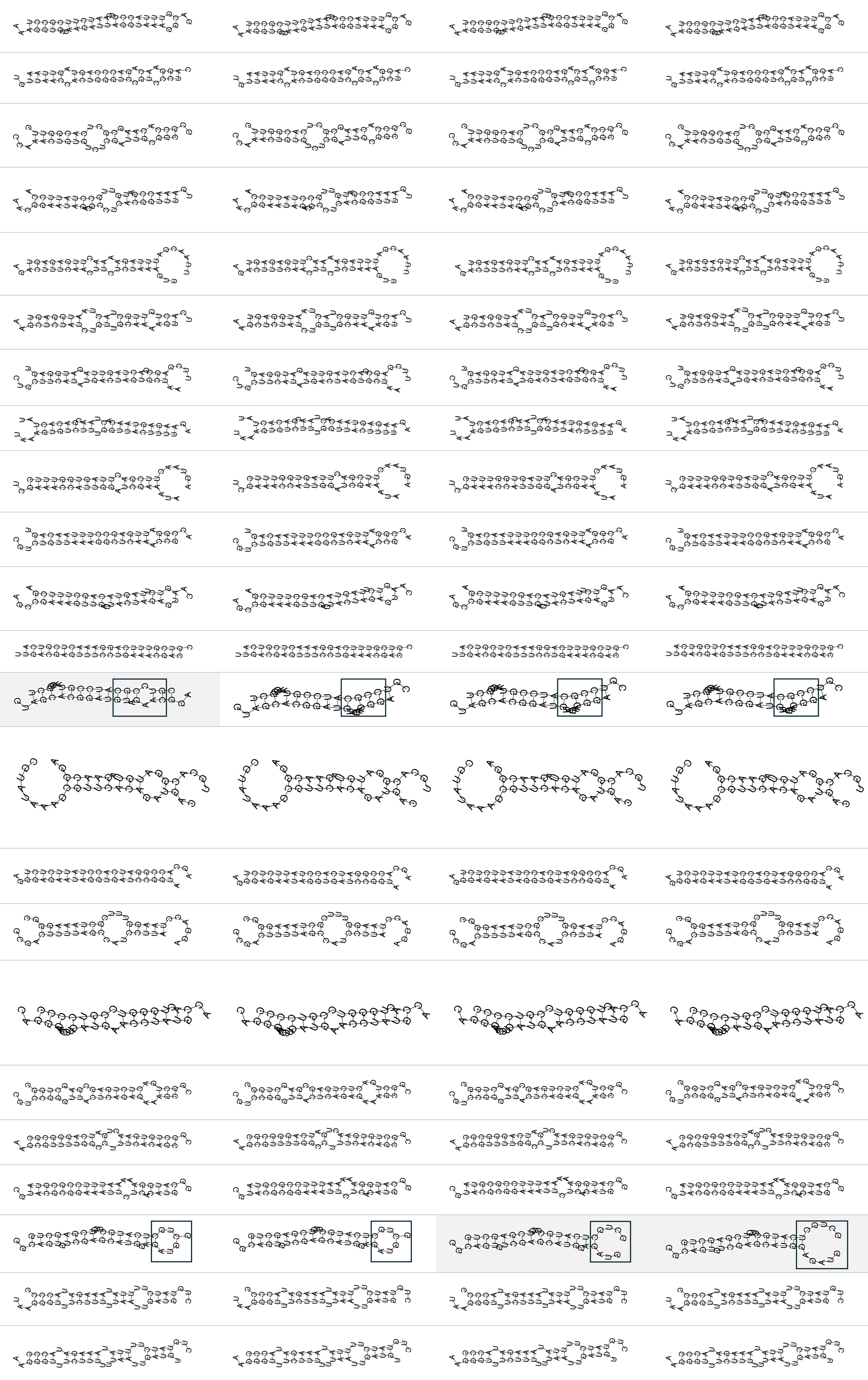

mir-788
mir-789-1
mir-790
mir-791
mir-792
mir-794
mir-795
mir-797
mir-79
mir-800
mir-80
mir-8186
mir-8187
mir-8188
mir-8189
mir-8190
mir-8191
mir-8192
mir-8193
mir-8194
mir-8195
mir-8196a
mir-8196b

mir-8197
mir-8198
mir-8199
mir-81
mir-8200
mir-8201
mir-8202
mir-8203
mir-8204
mir-8205
mir-8206
mir-8207
mir-8208
mir-8209
mir-8210
mir-8211
mir-8212
mir-82
mir-83
mir-84
mir-85
mir-86
mir-87
mir-90

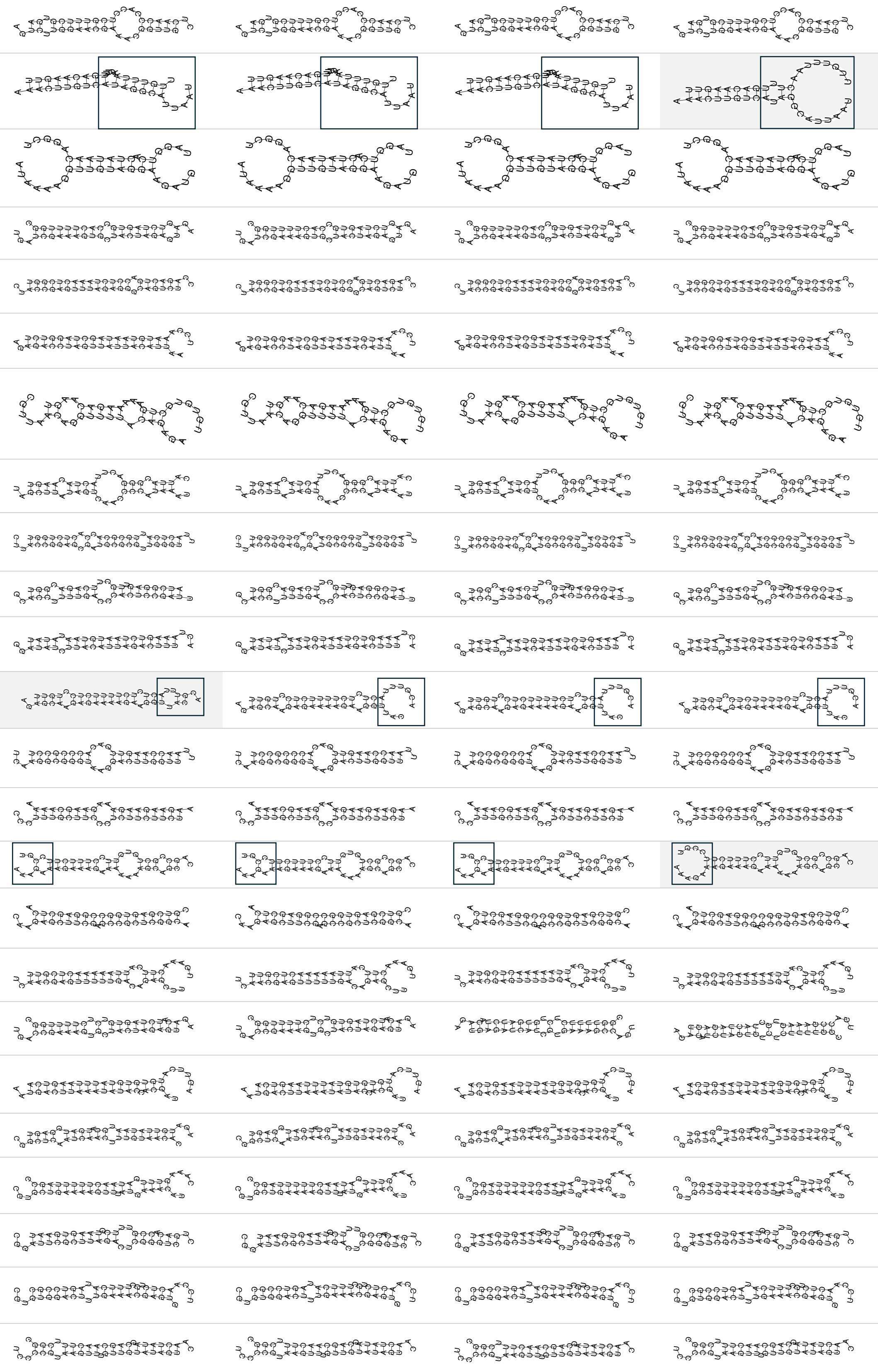

Table S3: *C. elegans* miRNA duplexes arising from precursor hairpins (constrained) compared to minimal free energy (MFE) duplex structures at 20°C

miRNA	Constrained Duplex (20°C)	MFE Duplex (20°C)
let-7	`          U           UU`<br>`  UGAGGUAG AGGUUGUAUAG`<br>`  AUUCCAUC UUUAACGUAUC`<br>`CC        U`	`          U           UU`<br>`  UGAGGUAG AGGUUGUAUAG`<br>`  AUUCCAUC UUUAACGUAUC`<br>`CC        U`
lin-4	`   U   U    C    A    GA`<br>`    CCC GAGA CUCA GUGU`<br>`    GGG CUCU GGGU CACA`<br>`CCAU   C    C    C`	`   U   U    C    A    GA`<br>`    CCC GAGA CUCA GUGU`<br>`    GGG CUCU GGGU CACA`<br>`CCAU   C    C    C`
lsy-6	`             A   U     U`<br>`   AAAUGCGUCU GUA CAAAA`<br>`   UUUACGCAGA UAU GUUUU`<br>`AGC          G   -`	`             A   U     U`<br>`   AAAUGCGUCU GUA CAAAA`<br>`   UUUACGCAGA UAU GUUUU`<br>`AGC          G   -`
mir-1019	`    U     U   UC     U  UUUU`<br>`   G GAGCA UGU  GAGUU CA`<br>`   C UUCGU ACA  CUUAA GU`<br>`GAC U     U   C-     U  C`	`    U     U   UC     U  UUUU`<br>`   G GAGCA UGU  GAGUU CA`<br>`   C UUCGU ACA  CUUAA GU`<br>`GAC U     U   C-     U  C`
mir-1020	`   U                 CUU`<br>`  G AAGUGUUACAGAAUAAU`<br>`  C UUCACAGUGUCUUAUUA`<br>`GA U`	`   U                 CUU`<br>`  G AAGUGUUACAGAAUAAU`<br>`  C UUCACAGUGUCUUAUUA`<br>`GA U`
mir-1022	`  AA          A    GC-   C`<br>`    GAUCAUUGUU GGAC   CAU`<br>`    CUAGUAGUAA CCUG   GUA`<br>`CGAC          -    AUA`	`  AA          A    GC-   C`<br>`    GAUCAUUGUU GGAC   CAU`<br>`    CUAGUAGUAA CCUG   GUA`<br>`CGAC          -    AUA`
mir-124	`      C    CUA   A     GU`<br>`  GCAU CACC   GUG CUUUA`<br>`  CGUA GUGG   CAC GGAAU`<br>`AC    A    CG-   -`	`      C    CUA   A     GU`<br>`  GCAU CACC   GUG CUUUA`<br>`  CGUA GUGG   CAC GGAAU`<br>`AC    A    CG-   -`
mir-1819	`  A    U      AA      G CA`<br>`   AUCA GCUCAA  CAUUC A`<br>`   UAGU CGAGUU  GUAAG U`<br>`AGG    U      A-      G`	`  A    U      AA      GACA`<br>`   AUCA GCUCAA  CAUUC`<br>`   UAGU CGAGUU  GUAAG`<br>`AGG    U      A-      GU`
mir-1820	`             UU     AU   CG`<br>` UUUUGAUUGUUU  CGAUG  GUU`<br>` GAAACUAACAAA  GUUAC  CAA`<br>`A            U-     --`	`             UU     AUGUUCG`<br>` UUUUGAUUGUUU  CGAUG`<br>` GAAACUAACAAA  GUUAC`<br>`A            U-     CAA`
mir-1821	`      C    UGC      UU   AU`<br>`  UGCC AACU   AGACU  UCA`<br>`  AUGG UUGA   UCUGG  AGU`<br>`AG    A    UAU      --`	`      C    UGC       UCAAU`<br>`  UGCC AACU   AGACUUU`<br>`  AUGG UUGA   UCUGGAG`<br>`AG    A    UAU       U`
mir-1822	`       C    -  AA   UA  GGC`<br>`  AGUUU UCUG GG  AGC  UC`<br>`  UCAAA AGAC CC  UCG  AG`<br>`UC     A    U  CG   --`	`       C    -  AA    AUCGGC`<br>`  AGUUU UCUG GG  AGCU`<br>`  UCAAA AGAC CC  UCGA`<br>`UC     A    U  CG    G`
mir-1823	`    C     C -  A G     UUU`<br>`  AC CCUAA C CU U CAGUA`<br>`  UG GGAUU G GA A GUCAU`<br>`AA  A     U U  - G`	`    C     C -  A G     UUU`<br>`  AC CCUAA C CU U CAGUA`<br>`  UG GGAUU G GA A GUCAU`<br>`AA  A     U U  - G`
mir-1824	`  U  C       CU   --     UU`<br>`   GG AGUGUUU  CCC  CCAAC`<br>`   CC UCACAAG  GGG  GGUUG`<br>`CCG  U       U-   CC`	`  U  C       CU   --     UU`<br>`   GG AGUGUUU  CCC  CCAAC`<br>`   CC UCACAAG  GGG  GGUUG`<br>`CCG  U       U-   CC`
mir-1829a	`  A      C       U   U UA`<br>`   AGGGGA UUCUAAU GUU G`<br>`   UCUCUU AAGGUUA CAA C`<br>`UUA      U       C   -`	`  A      C       U   UGUA`<br>`   AGGGGA UUCUAAU GUU`<br>`   UCUCUU AAGGUUA CAA`<br>`UUA      U       C   C`
mir-1829b	`  A  C  UC      A      UA`<br>`   AG GA  UUCUAG UGGUUG`<br>`   UC CU  AAGGUC ACCAAC`<br>`UUA  U  UU      -`	`  A  C  UC      A      UA`<br>`   AG GA  UUCUAG UGGUUG`<br>`   UC CU  AAGGUC ACCAAC`<br>`UUA  U  UU      -`
mir-1829c	`  A  C       A  A      UA`<br>`   AG GAAAUUC AG UGGUUG`<br>`   UC CUUUAAG UC ACCAAC`<br>`UUA  U       G  -`	`  A  C       A  A      UA`<br>`   AG GAAAUUC AG UGGUUG`<br>`   UC CUUUAAG UC ACCAAC`<br>`UUA  U       G  -`
mir-1830	`      GU   A           CC`<br>`  CGAG  UUC CGUUUUCUAGG`<br>`  GCUC  AAG GUAAAGGAUCC`<br>`CG    AA   A`	`      GU   A           CC`<br>`  CGAG  UUC CGUUUUCUAGG`<br>`  GCUC  AAG GUAAAGGAUCC`<br>`CG    AA   A`
mir-1832a	`    G       AA         CU`<br>`  CA CGAUUCG  CUCCGCCCA`<br>`  GU GCUAAGC  GAGGCGGGU`<br>`UA  A       --`	`    G       AA         CU`<br>`  CA CGAUUCG  CUCCGCCCA`<br>`  GU GCUAAGC  GAGGCGGGU`<br>`UA  A       --`
mir-1832b	`               G       UU`<br>`  CAGCGAAUCGCUC GCCCACU`<br>`  GUCGCUUAGCGAG CGGGUGA`<br>`UA             A`	`               G       UU`<br>`  CAGCGAAUCGCUC GCCCACU`<br>`  GUCGCUUAGCGAG CGGGUGA`<br>`UA             A`
mir-1	`          C      GC   UA`<br>`  CAUACUUC UUACAU  CCA`<br>`  GUAUGAAG AAUGUA  GGU`<br>`AU        A      A-`	`          C      GC   UA`<br>`  CAUACUUC UUACAU  CCA`<br>`  GUAUGAAG AAUGUA  GGU`<br>`AU        A      A-`
mir-2207	`      A      A        AAG`<br>`  UGUG AUUGAG CUGUGUAU`<br>`  ACAC UAACUC GACACGUA`<br>`AC    G      G`	`      A      A        AAG`<br>`  UGUG AUUGAG CUGUGUAU`<br>`  ACAC UAACUC GACACGUA`<br>`AC    G      G`
mir-2208a	`           C       A   CC`<br>`  AAGUGUACC GAAUCUG UAU`<br>`  UUCAUAUGG CUUUGAC GUA`<br>`AC         U       -`	`           C       AUAUCC`<br>`  AAGUGUACC GAAUCUG`<br>`  UUCAUAUGG CUUUGAC`<br>`AC         U       GUA`
mir-2208b	`           C       A   CC`<br>`  AAGUGUACC GGAUCUG UAU`<br>`  UUCACAUGG UUUAGAC GUA`<br>`AC         U       -`	`           C       AUAUCC`<br>`  AAGUGUACC GGAUCUG`<br>`  UUCACAUGG UUUAGAC`<br>`AC         U       GUA`

mir-2209a

```
  –         A  CU    C UC
 G AGUGUAACC CU  UCUC U
 C UCACAUUGG GA  AGAG A
A A         C  CU    –

  –         A  CU    CUUC
 G AGUGUAACC CU  UCUC
 C UCACAUUGG GA  AGAG
A A         C  CU    A
```

mir-2209b

```
           AA    U    C UC
   AGUGUAAC  CUC UCUC U
   UCGUGUUG  GAG AGAG A
ACU        GC    U    –

           AA    U    CUUC
   AGUGUAAC  CUC UCUC
   UCGUGUUG  GAG AGAG
ACU        GC    U    A
```

mir-2209c

```
  –          CAC     G UU
 G AGUGUAACCG   GUCUU U
 C UCACAUUGGC   CAGAA A
A A          CAC     A

  –          CAC     GUUU
 G AGUGUAACCG   GUCUU
 C UCACAUUGGC   CAGAA
A A          CAC     AA
```

mir-2210

```
  A  C   U     AAU     GG
   GG AGA CAAUC   UUUUA
   CC UCU GUUAG   GAAAU
CAC  A   C     CU–

  A  C   U     A     UAGG
   GG AGA CAAUC AUUUU
   CC UCU GUUAG UGAAA
CAC  A   C     C     U
```

mir-2211

```
  C    A    UUC  C      CG
   CUCC UCUA   UC AUCUGA
   GAGG AGAU   AG UGGACU
AAA    –    UUA  A

  C    A    UUC  C      CG
   CUCC UCUA   UC AUCUGA
   GAGG AGAU   AG UGGACU
AAA    –    UUA  A
```

mir-2212

```
      AG    UAG   U    UG
  UGGC  AUCA   GCU ACUU
  ACCG  UAGU   CGG UGAA
CU    AA    UUA   –

      AG    UAG   U    UG
  UGGC  AUCA   GCU ACUU
  ACCG  UAGU   CGG UGAA
CU    AA    UUA   –
```

mir-2213

```
  U     A–     C       GA
   GGCGG  CUCUU ACAGUUU
   CCGUC  GAGAA UGUCGAA
AAU     AG     –

  U     A–     C       GA
   GGCGG  CUCUU ACAGUUU
   CCGUC  GAGAA UGUCGAA
AAU     AG     –
```

mir-2215

```
                   G  CC
  ACAGCACGUGUUACGAU CU
  UGUUGUGCGCGAUGCUA GA
UU                 A

                   G  CC
  ACAGCACGUGUUACGAU CU
  UGUUGUGCGCGAUGCUA GA
UU                 A
```

mir-2216

```
               C     GC
  GCACAUUUUAAGU GGUAG
  CGUGUAAAAUUCA CUAUC
UC             U

               C     GC
  GCACAUUUUAAGU GGUAG
  CGUGUAAAAUUCA CUAUC
UC             U
```

mir-2217a

```
   A         GUC  U     UC
  C GAGUGGGCA   GG GUCGA
  G UUUGUCCGU   CC CAGCU
UG C         GUU  –

   A         GUC  U     UC
  C GAGUGGGCA   GG GUCGA
  G UUUGUCCGU   CC CAGCU
UG C         GUU  –
```

mir-2217b

```
   A         GUC  U     UC
  C GAGUGGGCA   GG GUCAA
  G UUUGUUCGU   CC CAGUU
CG C         GUU  –

   A         GUC  U     UC
  C GAGUGGGCA   GG GUCAA
  G UUUGUUCGU   CC CAGUU
CG C         GUU  –
```

mir-2218a

```
           AGU   AA    CA
  CAAACUACA   UUU  GCCU
  GUUUGAUGU   AAG  CGGA
AU         GAU   AC

           AGU   AA    CA
  CAAACUACA   UUU  GCCU
  GUUUGAUGU   AAG  CGGA
AU         GAU   AC
```

mir-2218b

```
  AG             UC    UC
    ACUACAAACUACA  AUUU
    UGAUGUUUGAUGU  UAAA
AGAG             U–

  AG             UC    UC
    ACUACAAACUACA  AUUU
    UGAUGUUUGAUGU  UAAA
AGAG             U–
```

mir-2219

```
ACA              A
   GCUUUCUCUCGCAC UCGUC
   CGAAAGGGAGCGUG AGCAG
                 A     UC

ACA              A
   GCUUUCUCUCGCAC UCGUC
   CGAAAGGGAGCGUG AGCAG
                 A     UC
```

mir-2220

```
       A        U   U UC
  GUAAG CCAUAAAC AUU A
  CAUUC GGUGUUUG UAA U
GA     A        U   C

       A        U   UAUC
  GUAAG CCAUAAAC AUU
  CAUUC GGUGUUUG UAA
GA     A        U   CU
```

mir-228

```
  A    C    C    A   A GG
   AUGG ACUG AUGA UUC C
   UACC UGGC UACU AGG G
CGC    A    A    –   C

  A    C    C    A   A GG
   AUGG ACUG AUGA UUC C
   UACC UGGC UACU AGG G
CGC    A    A    –   C
```

mir-229

```
 A        G U        CCA CG
  AUGACACU G UAUCUUUU   U
  UACUGUGG C AUGGAAAG   A
AG        G U        –––

 A        G U        CCAUCG
  AUGACACU G UAUCUUUU
  UACUGUGG C AUGGAAAG
AG        G U        A
```

mir-230

```
  A        –      U     UA
   CUUGGUCG GCGAUU AAUAU
   GGACCAGC UGUUGA UUAUG
AGA        G      –

  A        –      U     UA
   CUUGGUCG GCGAUU AAUAU
   GGACCAGC UGUUGA UUAUG
AGA        G      –
```

mir-231

```
     A     ––   AC      UA
  CUG CUGUU  UCA  AGCUUG
  GAC GACAA  AGU  UCGAAU
AA   G     CU   GC

     A     ––   AC      UA
  CUG CUGUU  UCA  AGCUUG
  GAC GACAA  AGU  UCGAAU
AA   G     CU   GC
```

mir-232

```
  C        UC   AU    UC
   CUGCAGUU  GAUG  UUUA
   GGCGUCAA  CUAC  AAAU
AGU        UU   GU

  –        UC   AU    UC
 C CUGCAGUU  GAUG  UUUA
 G GGCGUCAA  CUAC  AAAU
A U        UU   GU
```

mir-233

```
      C   – C        C  UA
  UCGC CAU C CGUUGCUC AA
  GGCG GUA G GUAACGAG UU
AG    U   C C        –

      C   – C        CAAUA
  UCGC CAU C CGUUGCUC
  GGCG GUA G GUAACGAG
AG    U   C C        UU
```

mir-234

```
  C       CA    U     UA
   GGUAUUC  GAGU GAUAA
   CCAUAAG  CUCG UUAUU
UUC       AG    –

  C       CA    U     UA
   GGUAUUC  GAGU GAUAA
   CCAUAAG  CUCG UUAUU
UUC       AG    –
```

mir-235

```
       UU  CU  U     AAUU
  AGGCC  GG  GA UGCAA
  UCCGG  CC  CU ACGUU
AG     C–  CU  C     AU

       UU  CU  U     AAUU
  AGGCC  GG  GA UGCAA
  UCCGG  CC  CU ACGUU
AG     C–  CU  C     AU
```

mir-236

```
      –       UU  A    UAGA
  CGUC UUACCUG  CA UAUU
  GCAG AAUGGAC  GU AUAA
UC    U       U–  C    U

      –       UU  A    UAGA
  CGUC UUACCUG  CA UAUU
  GCAG AAUGGAC  GU AUAA
UC    U       U–  C    U
```

mir-237

```
     C   G  UU     A   CU
  UCC UGA AA  CUCGA CAG
  AGG ACU UU  GAGCU GUC
CC   A   G  UU     –

     C   G  UU     A   CU
  UCC UGA AA  CUCGA CAG
  AGG ACU UU  GAGCU GUC
CC   A   G  UU     –
```

mir-238
```
        U C     C  U    GC                  U C     C  U    GC
  UGGAUG U UCGGA GU CAAA              UGGAUG U UCGGA GU CAAA
  ACUUAC G AGCCU CA GUUU              ACUUAC G AGCCU CA GUUU
AG      C U     -  U              AG      C U     -  U
```

mir-239a
```
           C  A        GG                    C  A        GG
  UUUGUACUA AC UAGGUACU               UUUGUACUA AC UAGGUACU
  AAACGUGAU UG AUCUGUGA               AAACGUGAU UG AUCUGUGA
AC         C  -                   AC         C  -
```

mir-239b
```
         UA         A UG                   UA         A UG
  UUUGUAC  CACAAAAGU C                UUUGUAC  CACAAAAGU C
  AAACGUG  GUGUUUUCA G                AAACGUG  GUGUUUUCA G
AA       UG         C             AA       UG         C
```

mir-240
```
           U A A    A UGC                    U A A    AAUGC
  CGAGGAUUU G G CUAG A                CGAGGAUUU G G CUAG
  GCUUCUAAA C C GGUC U                GCUUCUAAA C C GGUC
UC         C C C    A             UC         C C C    AU
```

mir-241
```
          G  C    -   GA                    G  C    AAUGA
  UGAGGUAG UG GAGA AAU                UGAGGUAG UG GAGA
  ACUUCGUC AC CUCU UUA                ACUUCGUC AC CUCU
CU        G  U    G               CU        G  U    GUUA
```

mir-243
```
         G                                 G
  UAUCUCG UGCGAUCGUAC                 UAUCUCG UGCGAUCGUAC
  AUAGGGC GCGCUAGCAUG                 AUAGGGC GCGCUAGCAUG
CU       G           GC           CU       G           GC
```

mir-244
```
           UAC         UG                    UAC         UG
 UCUUUGGUUG   AAAGUGGUA              UCUUUGGUUG   AAAGUGGUA
 GGAAAUCGAC   UUUCGUCAU              GGAAAUCGAC   UUUCGUCAU
A          U--                    A          U--
```

mir-245
```
          C A  U   U   UG                   C A  U   UAAUUG
  GCUAUUUG A GG ACC AAU               GCUAUUUG A GG ACC
  CGAUGAAC U CC UGG UUA               CGAUGAAC U CC UGG
CU        C C  C   -              CU        C C  C   UUA
```

mir-246
```
  G    A   UUGU       UA           CG    A   UUGU       UA
 C CCUA CCG    CAUGUAA               CCUA CCG    CAUGUAA
 G GGAU GGC    GUACAUU               GGAU GGC    GUACAUU
C A    G   UUU-                   CGA    G   UUU-
```

mir-247
```
  U      A  U     A   CC            U      A  U     A   CC
   AGAGAA AG UUCUA UUA               AGAGAA AG UUCUA UUA
   UCUCUU UC GAGAU AGU               UCUCUU UC GAGAU AGU
UCU      A  C     C               UCU      A  C     C
```

mir-249
```
A       A    C   UU               A       A    C   UU
 GCAACGC CAAA GUC  CUGUG           GCAACGC CAAA GUC  CUGUG
 CGUUGCG GUUU CAG  GACAC           CGUUGCG GUUU CAG  GACAC
C       A    U   --     U         C       A    U   --     U
```

mir-250
```
   UU      C  C     CCG              UU      C  C     CCG
 CC  CAGUUG CU GUGAU               CC  CAGUUG CU GUGAU
 GG  GUCAAC GA CACUA               GG  GUCAAC GA CACUA
C  UU      U  -     A             C  UU      U  -     A
```

mir-252
```
AUAA    U  U  C C                 AUAA    U  U  C C
    GUAG AG GC G AGGUAA               GUAG AG GC G AGGUAA
    CGUC UC CG C UCCAUU               CGUC UC CG C UCCAUU
        U  -  U A      C                  U  -  U A      C
```

mir-253
```
           - A   C     CA                    - A   C     CA
  CUUUUCACA C CCU ACUAA               CUUUUCACA C CCU ACUAA
  GAAGGGUGU G GGA UGAUU               GAAGGGUGU G GGA UGAUU
GG         U C   -                GG         U C   -
```

mir-254
```
UACA AA  AU-      C  CA           UACAGAA  AU-      C  CA
    G  GC   AGAUUU CA                    GC   AGAUUU CA
    C  CG   UCUAAA GU                    CG   UCUAAA GU
     AG  CUU      C                   CAG  CUU      C
```

mir-255
```
               G     CUC                         G     CUC
  GUAAGAAAUCUUU UAGUU                 GUAAGAAAUCUUU UAGUU
  CAUUUUUUAGAGA GUCAA                 CAUUUUUUAGAGA GUCAA
GA             A     A            GA             A     A
```

mir-259
```
  A        -  U    U   A CA         A        -  U    U   AGCA
   AAUCUCAU CC AAUC GGU G            AAUCUCAU CC AAUC GGU
   UUAGGGUA GG UUAG CCA C            UUAGGGUA GG UUAG CCA
CAG        C  U    -   C          CAG        C  U    -   CC
```

mir-2953
```
          U   C    AAAU                     U   C    AAAU
  UACAGAAG GUU GUGA                   UACAGAAG GUU GUGA
  AUGUCUUC CGA CACU                   AUGUCUUC CGA CACU
CG        U   U    AG             CG        U   U    AG
```

mir-2
```
            -   G  U    UG                    -   G  U    UG
   CAUCAAAGC GGU GU GAUG             CAUCAAAGC GGU GU GAUG
   GUAGUUUCG CCG CA CUAU             GUAGUUUCG CCG CA CUAU
CGU         A   A  -              CGU         A   A  -
```

mir-34
```
   A       U  U      G UG            A       U  U      GUUG
    GGCAGUG GG UAGCUG U               GGCAGUG GG UAGCUG
    CCGUCAC UC AUCGGC A               CCGUCAC UC AUCGGC
CCCA       U  C      -            CCCA       U  C      A
```

mir-354
```
   U     U       -   AU              U     U       -   AU
 GG GCGGC GCAGACG GGU              GG GCGGC GCAGACG GGU
 CC CGUCG UGUUUGU CCA              CC CGUCG UGUUUGU CCA
U  U     U       U                U  U     U       U
```

mir-355
```
   U         CU  -    UG            UU         CU  -    UG
  U UGUUUUAGC  GA GCUA                UGUUUUAGC  GA GCUA
  G ACAAAAUCG  CU CGAU                ACAAAAUCG  CU CGAU
CC U         UU  U                CCGU         UU  U
```

mir-356b
```
      -   AC   U  U      U              -   AC   U  U      U
  UGGU GAG  ACG CG AACGAA             UGGU GAG  ACG CG AACGAA
  ACCA CUC  UGC GC UUGUUU             ACCA CUC  UGC GC UUGUUU
AC    A   GU   -  -               AC    A   GU   -  -
```

mir-357
```
  C   A     -   C    A GC           C   A     -   C    AUGC
   CCU CAACG CUG GCAU U              CCU CAACG CUG GCAU
   GGA GUUGC GAC CGUA A              GGA GUUGC GAC CGUA
UGA   C     U   -    A            UGA   C     U   -    AA
```

mir-358
```
      G C    C  U    CUGU               G C    C  U    CUGU
  ACCU G CAGG AU CCAA                 ACCU G CAGG AU CCAA
  UGGA C GUCC UA GGUU                 UGGA C GUCC UA GGUU
UC    A U    C  U    A            UC    A U    C  U    A
```

mir-35

```
             U  A A    UA
  UGCUGGUUUCU CC C GUGG
  ACGAUCAAAGG GG G CACU
UG           U  - C
```

```
             U  A A    UA
  UGCUGGUUUCU CC C GUGG
  ACGAUCAAAGG GG G CACU
UG           U  - C
```

mir-360

```
      C  UU
UUGUGA CG  GUUACGGUCA
AACACU GC  UAAUGCCAGU
      U  CC
```

```
      C  UU
UUGUGA CG  GUUACGGUCA
AACACU GC  UAAUGCCAGU
      U  CC
```

mir-36

```
  C  C           CA   CUA
   GC AAUUUUCGCUU  GUG
   CG UUAAAAGUGGG  CAC
GUA  C           C-   U
```

```
  C  C           CA   CUA
   GC AAUUUUCGCUU  GUG
   CG UUAAAAGUGGG  CAC
GUA  C           C-   U
```

mir-37

```
           C    G     CUA
  UGUGGGUGU CGUU CGGUG
  ACGUUCACA GUGG GCCAC
UG         A    -     U
```

```
           C    G     CUA
  UGUGGGUGU CGUU CGGUG
  ACGUUCACA GUGG GCCAC
UG         A    -     U
```

mir-38

```
             C G      UA
  UCCGGUUUUUU C UGGUGA
  AGGUCAAAAAG G GCCACU
UG           A G
```

```
             C G      UA
  UCCGGUUUUUU C UGGUGA
  AGGUCAAAAAG G GCCACU
UG           A G
```

mir-392

```
   G   U         G    U
  A CAU CGUGGUUGA GAUA
  U GUG GCACUAGCU CUAU
AG A   U         A
```

```
  AG   U         G    U
    CAU CGUGGUUGA GAUA
    GUG GCACUAGCU CUAU
AGUA   U         A
```

mir-39

```
           -  U      A UA
  AGCUGAUUU CG CUUGGU A
  UCGACUAAA GU GGGCCA U
GU         U  -      C
```

```
           -  U      AAUA
  AGCUGAUUU CG CUUGGU
  UCGACUAAA GU GGGCCA
GU         U  -      CU
```

mir-40

```
     G          A  A   UA
  AGU GAUGUAUGCC UG UGA
  UCG CUACAUGUGG GC ACU
AA   A          -  C
```

```
     G          A  A   UA
  AGU GAUGUAUGCC UG UGA
  UCG CUACAUGUGG GC ACU
AA   A          -  C
```

mir-41

```
             U  GCA     UA
  GGUGGUUUUUC CU   GUGA
  CCACUAAAAAG GG   CACU
AU           U  GC-
```

```
             -    CA     UA
  GGUGGUUUUUC UCUG  GUGA
  CCACUAAAAAG GGGC  CACU
AU           U    --
```

mir-42

```
            U   UUUU      AG
   GUGGGUGUU GCU    CGGUGA
   CAUCUACAA UGG    GCCACU
AGA         U   ----
```

```
            U   UUUU      AG
   GUGGGUGUU GCU    CGGUGA
   CAUCUACAA UGG    GCCACU
AGA         U   ----
```

mir-43

```
      U    -     A     UAUG
  GACA CAAG AAACU GUGAU
  CUGU GUUC UUUGA CACUA
CG    C    A     -     U
```

```
      U    -     A     UAUG
  GACA CAAG AAACU GUGAU
  CUGU GUUC UUUGA CACUA
CG    C    A     -     U
```

mir-44

```
           C  G       UA
  CUGGAUGUG UC UUGGUCA
  GACUUACAC AG GAUCAGU
UC         -  A
```

```
           C  G       UA
  CUGGAUGUG UC UUGGUCA
  GACUUACAC AG GAUCAGU
UC         -  A
```

mir-45

```
           C  G       UA
  CUGGAUGUG UC UUAGUCA
  GACUUACAC AG GAUCAGU
UC         -  A
```

```
           C  G       UA
  CUGGAUGUG UC UUAGUCA
  GACUUACAC AG GAUCAGU
UC         -  A
```

mir-46

```
          CG-     U    GU
  AAGAGAGC   UCUAU GACA
  UUCUCUCG   AGGUA CUGU
AC        CUG     -
```

```
          CG-     U    GU
  AAGAGAGC   UCUAU GACA
  UUCUCUCG   AGGUA CUGU
AC        CUG     -
```

mir-47

```
          A    AU-     GU
  AAGAGAGC GUCU   UGACA
  UUCUCUCG CGGA   ACUGU
AC        -    GGU
```

```
          A    AU-     GU
  AAGAGAGC GUCU   UGACA
  UUCUCUCG CGGA   ACUGU
AC        -    GGU
```

mir-4805

```
       C      G C      GC
  UGCGG AAAUUU C GAAUUU
  ACGCC UUUAGA G UUUAAA
AC     U      - U
```

```
       C      G C      GC
  UGCGG AAAUUU C GAAUUU
  ACGCC UUUAGA G UUUAAA
AC     U      - U
```

mir-4806

```
   A              C  GC
  C CUUACCGGCUGAGC AU
  G GAAUGGCCGACUCG UA
CG A              A
```

```
  CA              CAUGC
    CUUACCGGCUGAGC
    GAAUGGCCGACUCG
CGGA              AUA
```

mir-4808

```
        U   U   U    UC
  GUAAGA AGA UAG GCUU
  CAUUCU UCU AUC CGAA
GA      C   U   U
```

```
        U   U   U    UC
  GUAAGA AGA UAG GCUU
  CAUUCU UCU AUC CGAA
GA      C   U   U
```

mir-4809

```
   U         -    A    UC
  G AAGUUCAGA GUUG AUUA
  C UUCGGGUCU CAAC UAAU
GA C         U    A
```

```
   U         -    A    UC
  G AAGUUCAGA GUUG AUUA
  C UUCGGGUCU CAAC UAAU
GA C         U    A
```

mir-4810b

```
         C     -      UAU
  GUAGGUU AUGAG UAGUCA
  CAUUCAA UACUC AUCAGU
GA       C     U
```

```
         C     -      UAU
  GUAGGUU AUGAG UAGUCA
  CAUUCAA UACUC AUCAGU
GA       C     U
```

mir-4811

```
            CU      A AG
  UGAACAAUAC  GUGUUA A
  GCUUGUUAUG  CACAAU U
UC          CU      A
```

```
  U         CU      AAAG
   GAACAAUAC  GUGUUA
   CUUGUUAUG  CACAAU
UCG         CU      AU
```

mir-4812

```
 A    C C        A    U
  AGAG G UUGUAGUG GUUG
  UCUC U AACGUCAC CAAC
UA    U U        -
```

```
 A    C C        A    U
  AGAG G UUGUAGUG GUUG
  UCUC U AACGUCAC CAAC
UA    U U        -
```

mir-4813

```
           A  AA        CU
  AGACUAUCU GG  AUAUUGAA
  UCUGGUAGG CC  UAUAACUU
GG         -  A-
```

```
           A  AA        CU
  AGACUAUCU GG  AUAUUGAA
  UCUGGUAGG CC  UAUAACUU
GG         -  A-
```

mir-4814

```
                  G   CU
  UUCUCAACCAACUUUG CCA
  AAGAGUUGGUUGAAAC GGU
UG                G
```

```
                  G   CU
  UUCUCAACCAACUUUG CCA
  AAGAGUUGGUUGAAAC GGU
UG                G
```

mir-4816

```
         - UUU-         CAA
  GUAAGUG G     UUGUAGAU
  CAUUCAC C     AACAUCUA
GA       G UUUU
```

```
         - UUU-         CAA
  GUAAGUG G     UUGUAGAU
  CAUUCAC C     AACAUCUA
GA       G UUUU
```

mir-48

```
      G      CA       CGA
  UGAG UAGGCU  GUAGAUG
  GCUC AUCCGA  CACCUAC
AC    G      A-       A
```

```
  U   G      CA       CGA
   GAG UAGGCU  GUAGAUG
   CUC AUCCGA  CACCUAC
ACG   G      A-       A
```

mir-49

```
  C       G     AU     CC
   GCAGUUU UUGUG  GUGCU
   CGUCGAA AGCAC  CACGA
AGA       G     --     A
```

```
  C       G     AU     CC
   GCAGUUU UUGUG  GUGCU
   CGUCGAA AGCAC  CACGA
AGA       G     --     A
```

mir-50

```
                 UCU    UU
  UGAUAUGUCUGGUAU   UGGG
  GCUAUGCAGAUUAUA   GCCC
CA               -C-
```

```
                 UCU    UU
  UGAUAUGUCUGGUAU   UGGG
  GCUAUGCAGAUUAUA   GCCC
CA               -C-
```

mir-51

```
      C   G  C  A      UU
  UACC GUA CU CU UCCAUG
  GUGG CAU GA GA AGGUAC
AC    A   G  C  -
```

```
      C   G  C  A      UU
  UACC GUA CU CU UCCAUG
  GUGG CAU GA GA AGGUAC
AC    A   G  C  -
```

mir-52

```
  C    GUA   A   UUC    CU
   ACCC   CAU UGU   CGUG
   UGGG   GUA ACA   GCAC
CGA    AAA   -   UU-
```

```
  C    GUA   A   UUC    CU
   ACCC   CAU UGU   CGUG
   UGGG   GUA ACA   GCAC
CGA    AAA   -   UU-
```

mir-53

```
  C       CAU    UU     CU
   ACCCGUA   UUGU  CCGUG
   UGGGUAU   AACA  GGCAC
CGC       AU-    C-
```

```
  C       CAU    UU     CU
   ACCCGUA   UUGU  CCGUG
   UGGGUAU   AACA  GGCAC
CGC       AU-    C-
```

mir-54

```
  A    -    -  CG   A A CA
   GGAU AUGA GA  ACG G A
   CCUA UACU CU  UGC C U
GAG    A    U  AA   C A
```

```
  A    -    -  CG   AGAACA
   GGAU AUGA GA  ACG
   CCUA UACU CU  UGC
GAG    A    U  AA   CCAU
```

mir-5545

```
   C                 AA GC
  G CGGUUUGAUCUACAAAA  U
  C GCCAAACUAGAUGUUUU  A
UA A                 C-
```

```
   C                 AAUGC
  G CGGUUUGAUCUACAAAA
  C GCCAAACUAGAUGUUUU
UA A                 CA
```

mir-5546

```
      UUU   -         UCGU
  ACCC   CGC CAAUUUUUU
  UGGG   GCG GUUAAAAAG
GU    UAC   C
```

```
      UUU   -         UCGU
  ACCC   CGC CAAUUUUUU
  UGGG   GCG GUUAAAAAG
GU    UAC   C
```

mir-5547

```
            A  A      AU
  CAACUUUUAG CC AUAGGC
  GUUGAAAAUC GG UAUCCG
CU          C  C
```

```
            A  A      AU
  CAACUUUUAG CC AUAGGC
  GUUGAAAAUC GG UAUCCG
CU          C  C
```

mir-5548

```
  G       A UC     C    GG
   CCUUCUC C  UCCACGG GGUA
   GGAAGAG G  AGGUGUC CCGA
AGA       - UU     -
```

```
  G       A UC     C    GG
   CCUUCUC C  UCCACGG GGUA
   GGAAGAG G  AGGUGUC CCGA
AGA       - UU     -
```

mir-5549

```
   -UUGU             GU
  C     GAAAUUAACGUGA
  G     UUUUGGUUGUACU
UG UUGUU
```

```
   CUUGU             GU
        GAAAUUAACGUGA
        UUUUGGUUGUACU
UGGUUGUU
```

mir-5550

```
C        A           C
 CCCGCCCA GAUUUCAUUUG
 GGGCGGGU CUAAAGUAAAC
         G
```

```
C        A           C
 CCCGCCCA GAUUUCAUUUG
 GGGCGGGU CUAAAGUAAAC
         G
```

mir-5551

```
     A      U    UC     AU
  UGU AAUGGU GGAA  UGGU
  ACG UUACCA UCUU  ACCA
CU   A      U    UA
```

```
     A      U    UC     AU
  UGU AAUGGU GGAA  UGGU
  ACG UUACCA UCUU  ACCA
CU   A      U    UA
```

mir-5552

```
     A           A     CC
  UGU GUUUGUAGUCU GCAGA
  ACA CAAACAUCAGA CGUCU
AC   C           C
```

```
     A           A     CC
  UGU GUUUGUAGUCU GCAGA
  ACA CAAACAUCAGA CGUCU
AC   C           C
```

mir-5553

```
          C  C         UU
  UUGCCACG GC AUCCAUUGA
  AACGGUGC CG UGGGUAACU
AG        A  A
```

```
          C  C         UU
  UUGCCACG GC AUCCAUUGA
  AACGGUGC CG UGGGUAACU
AG        A  A
```

mir-5592

```
                       UG
  CGGCCCUUACCGUUUAAUACA
  GCCGGGAAUGGCAAAUUAUGU
CG
```

```
                       UG
  CGGCCCUUACCGUUUAAUACA
  GCCGGGAAUGGCAAAUUAUGU
CG
```

mir-5593

```
   A   C      AU      UC
  G UGG UGGAAU  CGGUAU
  C ACC ACCUUA  GCCAUA
AA A   A      C-
```

```
   A   C      AU      UC
  G UGG UGGAAU  CGGUAU
  C ACC ACCUUA  GCCAUA
AA A   A      C-
```

mir-5594

```
                 U    AU
  AAGAGUACUGUAGUU CAAA
  UUCUCAUGACAUCAA GUUU
AC               -
```

```
                 U    AU
  AAGAGUACUGUAGUU CAAA
  UUCUCAUGACAUCAA GUUU
AC               -
```

mir-5595

```
             U       CA CU
  UCUCUUUUUUC CGCAUGC  U
  AGAGAGAAGAG GUGUGCG  A
GC           -       AG
```

```
             U       - AUCU
  UCUCUUUUUUC CGCAUGC C
  AGAGAGAAGAG GUGUGCG G
GC           -       A A
```

mir-55

```
            C   -   U  UA
  CGGCAGAAAC UAU CGG UA
  GUCGUCUUUG AUA GCC AU
GA          A   U   C
```

```
            C   -   UUAUA
  CGGCAGAAAC UAU CGG
  GUCGUCUUUG AUA GCC
GA          A   U   CAU
```

mir-56

```
         UC    U     UGUACC
  UGGCGGA  CAUU UGGGU
  GUCGCCU  GUAA GCCCA
GA       UU    U     U
```

```
         UC    U     UGUACC
  UGGCGGA  CAUU UGGGU
  GUCGCCU  GUAA GCCCA
GA       UU    U     U
```

mir-57

```
      C     A  G    U   GU
  UACC UGUAG UC AGCU UGU
  GUGG ACAUC AG UCGA GCA
AC    A     -  A    -
```

```
      C     A  G    U   GU
  UACC UGUAG UC AGCU UGU
  GUGG ACAUC AG UCGA GCA
AC    A     -  A    -
```

mir-58a

```
      C    CUU  U      UC
  UGCC UACU   CG AUCUCA
  ACGG AUGA   GC UAGAGU
UA    C    CUU  -
```

```
      C    CUU  U      UC
  UGCC UACU   CG AUCUCA
  ACGG AUGA   GC UAGAGU
UA    C    CUU  -
```

mir-58b

```
     -       UG        UA
   GA CUCGGUG  UGAUCUCU
   CU GAGUUAC  ACUAGAGA
AAC  A       CA
```

```
     -       UG        UA
   GA CUCGGUG  UGAUCUCU
   CU GAGUUAC  ACUAGAGA
AAC  A       CA
```

mir-59
```
           -      AA   AA                      -      AA   AA
  UCGUCCUGA AAACGA  CGG                 UCGUCCUGA AAACGA  CGG
  AGUAGGACU UUUGCU  GCU                 AGUAGGACU UUUGCU  GCU
GU         A      AA                  GU         A      AA
```

mir-60
```
            -    C    A UC                      -    C    AAUC
  AACUGGAAGA GUGC AUAA A                AACUGGAAGA GUGC AUAA
  UUGAUCUUUU CACG UAUU U                UUGAUCUUUU CACG UAUU
AC          A    -    A               AC          A    -    AU
```

mir-61
```
       U    GG  U    CUU                     U    GG  U    CUU
  UGGGU ACGG  CU AGUC                   UGGGU ACGG  CU AGUC
  ACUCA UGCC  GA UCAG                   ACUCA UGCC  GA UCAG
CU     U    AA  -    U                CU     U    AA  -    U
```

mir-63
```
            --    A     CGU                     --    A     CGU
  UCUAACUCGU  CGGU GUCAU                UCUAACUCGU  CGGU GUCAU
  AGGUUGAGCG  GUCA CAGUA                AGGUUGAGCG  GUCA CAGUA
AA          AA    -     U             AA          AA    -     U
```

mir-64
```
     A      AG   --   CGAA                 A      AG     A - GAA
 UAUG CACUGA  CGU  UAC                 UAUG CACUGA  CGUU C C
 GUAC GUGACU  GCA  GUG                 GUAC GUGACU  GCAA G G
C    G      A-   AC                   C    G      A-    C U
```

mir-65
```
     A      AA    A C AA                   A      AA    A C AA
 UAUG CACUG  GCGUA C G                 UAUG CACUG  GCGUA C G
 GUAC GUGAC  CGCAU G C                 GUAC GUGAC  CGCAU G C
C    C     G-     C U                 C    C     G-     C U
```

mir-66
```
 CA        A        G GA               CA        A        GUGA
   UGACACUG UUAGGGAU U                   UGACACUG UUAGGGAU
   ACUGUGGC AAUCCUUA A                   ACUGUGGC AAUCCUUA
CAA        -        A                 CAA        -        AA
```

mir-67
```
  C    A     CC-      U UG              C    A     CC-      UAUG
   GCUC UUCUG   GGUUGU A                 GCUC UUCUG   GGUUGU
   UGAG AAGAU   CCAACA U                 UGAG AAGAU   CCAACA
AGA    A     CCU      C               AGA    A     CCU      CU
```

mir-70
```
  C         U     A     CA              C         U     AAUAACA
   GAAAUACUA CGACG AUAA                  GAAAUACUA CGACG
   CUUUGUGGU GCUGC UAAU                  CUUUGUGGU GCUGC
UAC         U     A                   UAC         U     AUAAU
```

mir-71
```
          U         G CG               U        U         GACG
 UGAAAGACA GGGUAGUGA A                  GAAAGACA GGGUAGUGA
 GCUUUUUGU CUUAUCACU U                  CUUUUUGU CUUAUCACU
C         -         A                 CG        -         AU
```

mir-72
```
  A    A     U   AU    GA               A    A     U   AU    GA
   GGCA GAUGU GGC  AGCU                  GGCA GAUGU GGC  AGCU
   CCGU UUACA CCG  UCGA                  CCGU UUACA CCG  UCGA
GCA    C     -   CU                   GCA    C     -   CU
```

mir-73
```
        U  -     GA     CAGC                  U  -     GA     CAGC
  UGGACU CC AUAUC  GCCA                 UGGACU CC AUAUC  GCCA
  ACUUGA GG UGUAG  CGGU                 ACUUGA GG UGUAG  CGGU
UG      C  A     AA                   UG      C  A     AA
```

mir-74
```
  C     U    C    UC   GC               C     U    C    U  CAGC
   GGGCU CCAU UCUU  CCA                  GGGCU CCAU UCUU CC
   UCUGA GGUA AGAA  GGU                  UCUGA GGUA AGAA GG
ACA     C    A    C-                  ACA     C    A    C U
```

mir-75
```
  C         CA     A  UA                C         CA     AAAUA
   AGUCGGUUG  AGCUU AA                   AGUCGGUUG  AGCUU
   UCGGCCAAC  UCGAA UU                   UCGGCCAAC  UCGAA
ACU         CA     A                  ACU         CA     AUU
```

mir-76
```
  U        -      U    UA               U        -      U    UA
   GGGCUUCA CAAUAG CGAA                  GGGCUUCA CAAUAG CGAA
   UCCGAAGU GUUGUU GCUU                  UCCGAAGU GUUGUU GCUU
AGU        A      -                   AGU        A      -
```

mir-77
```
            C     G   AU                          C     G   AU
  GAUGGUUGUG UCUGA GAA                  GAUGGUUGUG UCUGA GAA
  CUGUCGAUAC GGACU CUU                  CUGUCGAUAC GGACU CUU
AC          C     A                   AC          C     A
```

mir-784
```
      A    C GC        GA               U   A    C GC        GA
  UGGC CAAU U  GUACGUA                   GGC CAAU U  GUACGUA
  GUCG GUUG A  CAUGUAU                   UCG GUUG A  CAUGUAU
CC    C    U AA                       CCG   C    U AA
```

mir-785
```
  A         UU      A CA                A         UU      AACA
   GCACAGAAU  UUCGCU A                   GCACAGAAU  UUCGCU
   UGUGUUUUG  AAGUGA U                   UGUGUUUUG  AAGUGA
AGA         UU      A                 AGA         UU      AU
```

mir-786
```
  C        G          U CA              C        G          UACA
   GAAUAUCA UUGGGGUAUU A                 GAAUAUCA UUGGGGUAUU
   CUUGUAGU AGUCCCGUAA U                 CUUGUAGU AGUCCCGUAA
UAA        A          -               UAA        A          U
```

mir-787
```
          -AU-    U    CA                       -AU-    U    CA
  AAAGAUAC    ACGA CUUA                 AAAGAUAC    ACGA CUUA
  UUUCUAUG    UGCU GAAU                 UUUCUAUG    UGCU GAAU
GC        AUUU    C                   GC        AUUU    C
```

mir-788
```
       --      CU       G AG                 --      CU       G AG
  UCCGC  UUCUAA  UCCAUUU C              UCCGC  UUCUAA  UCCAUUU C
  AGGUG  AAGAUU  AGGUAAA G              AGGUG  AAGAUU  AGGUAAA G
AA     GC      --       G             AA     GC      --       G
```

mir-789-1
```
       A           A   C                     A           A   C
  AAUUG UGACCCAGACA GGA                 AAUUG UGACCCAGACA GGA
  UUAAC ACUGGGUCCGU CCU                 UUAAC ACUGGGUCCGU CCU
UG     C           C                  UG     C           C
```

mir-790
```
  C       UC-  G   A   CG               C       UC-  G   A   CG
   UUGGCAC   GC AAC CCG                  UUGGCAC   GC AAC CCG
   AACUGUG   CG UUG GGC                  AACUGUG   CG UUG GGC
CCA       UCU  A   C                  CCA       UCU  A   C
```

mir-791
```
  A       --  UU  A      GU             A       --  UU  A      GU
   CCUUAUC  CG  GU GCCAAA                CCUUAUC  CG  GU GCCAAA
   GGAAUAG  GC  CA CGGUUU                GGAAUAG  GC  CA CGGUUU
AAC       AC  CU  -                   AAC       AC  CU  -
```

mir-792
```
          C  A      AG   UU                     C  A      AGCAAUU
  UGAGAGUU AA AGAUUU  CAA               UGAGAGUU AA AGAUUU
  ACUUUCAA UU UCUAAA  GUU               ACUUUCAA UU UCUAAA
AG        C  C      --                AG        C  C      GUU
```

mir-794

```
         AU  U    G   CU
  UGAGGUA  CA CGUU UCA
  GCUCUAU  GU GCAA AGU
AA       CU  U    A

         AU  U    G   CU
  UGAGGUA  CA CGUU UCA
  GCUCUAU  GU GCAA AGU
AA       CU  U    A
```

mir-795

```
         G          G GCUU
  UGAGGUA AUUGAUCAGC A
  GCUUCAU UGACUAGUGC U
CU       A          - AA

  U      G           AGCUU
   GAGGUA AUUGAUCAGCG
   CUUCAU UGACUAGUGCU
CUG      A           AA
```

mir-797

```
  A      C  U A         GA
 U UCACAG AA C CAAUGAGAA
 A AGUGUC UU G GUUACUUUU
U A      -  U -

 UA      C  U A         GA
   UCACAG AA C CAAUGAGAA
   AGUGUC UU G GUUACUUUU
UAA      -  U -
```

mir-79

```
             C     CA  GA
  CUUUGGUGAUU AGCUU  AU
  GAAACCAUUGG UCGAA  UA
UC           A     A-

             C     CAAUGA
  CUUUGGUGAUU AGCUU
  GAAACCAUUGG UCGAA
UC           A     AUA
```

mir-800

```
  U               A   CA
   GACAAUUUCCGAGUU GGC
   CUGUUAAAGGCUCAA CCG
CGU               A

  U               A   CA
   GACAAUUUCCGAGUU GGC
   CUGUUAAAGGCUCAA CCG
CGU               A
```

mir-80

```
  A         --     U  G AC
   GCUUUCGAC  AUGAU CU A
   CGAAAGUUG  UACUA GA U
AGC         AU     -  G

  A         --     U  G AC
   GCUUUCGAC  AUGAU CU A
   CGAAAGUUG  UACUA GA U
AGC         AU     -  G
```

mir-8186

```
                      C
 ACUGCUCAAAGGACUUUGCUG
 UGACGAGUUUCCUGAAACGAC
U

                      C
 ACUGCUCAAAGGACUUUGCUG
 UGACGAGUUUCCUGAAACGAC
U
```

mir-8187

```
     GGAA       -----    GC
  UCG    UGCCUAC     GCCU
  AGC    ACGGAUG     CGGA
GU   ----       UAGAA

     GGAA       -----    GC
  UCG    UGCCUAC     GCCU
  AGC    ACGGAUG     CGGA
GU   ----       UAGAA
```

mir-8188

```
       AG     A   AG  ACGU
         GCAAG UGU  GC
         CGUUC ACA  UG
CGUAUAAAG     -   GA  AC

       AG     A   AG  ACGU
         GCAAG UGU  GC
         CGUUC ACA  UG
CGUAUAAAG     -   GA  AC
```

mir-8189

```
                     CGA
  UCUCUUAUCCACUAGGCCA
  GGAGAAUAGGUGAUCCGGU
AG                   A

                     CGA
  UCUCUUAUCCACUAGGCCA
  GGAGAAUAGGUGAUCCGGU
AG                   A
```

mir-8190

```
  CG        CUUU     CCAGGA
    GGAAAUCG    GGAAU
    CUUUUAGC    CCUUA
GCGA        CAU-

  CG        CUUU     CCAGGA
    GGAAAUCG    GGAAU
    CUUUUAGC    CCUUA
GCGA        CAU-
```

mir-8191

```
     -----    C     C  CA
  CCC     CUGC UGGGU AC
  GGG     GAUG ACCUA UG
CA   AACCU    A     -

     -----    C     C  CA
  CCC     CUGC UGGGU AC
  GGG     GAUG ACCUA UG
CA   AACCU    A     -
```

mir-8192

```
  C    G  C       AG   GC
   GGUC AG GAGUCUC  UCG
   CCGG UU CUCAGAG  AGC
CGU    G  A       AA

  C    G  C       AG   GC
   GGUC AG GAGUCUC  UCG
   CCGG UU CUCAGAG  AGC
CGU    G  A       AA
```

mir-8193

```
           A UC       GC
  CGCGGGACU G  AAGUGUCG
  GCGUUUUGG C  UUCACAGC
AA         C U-

           A UC       GC
  CGCGGGACU G  AAGUGUCG
  GCGUUUUGG C  UUCACAGC
AA         C U-
```

mir-8194

```
              AA  -    GG
  AUGCGCCUUUAA  AG GUAC
  UACGCGGAAAUU  UC CAUG
CG            C-  A

              AA  -    GG
  AUGCGCCUUUAA  AG GUAC
  UACGCGGAAAUU  UC CAUG
CG            C-  A
```

mir-8195

```
      -    -  -  G-   UCGUCG
  GUCG AGCU GU CC  UAC
  CAGU UCGA CA GG  AUG
GG    G    G  U  AG

      -    GUC     -  GU G
  GUCG AGCU   CGUAC UC  C
  CAGU UCGA   GCAUG AG  G
GG    G    ---     G  AU
```

mir-8196a

```
  C    U     U   UU    GUC
   CCCA AGAAA AUU  CUAU
   GGGU UCUUU UAA  GAUG
UAA    U     U   UU

  C    U     U   UU    GUC
   CCCA AGAAA AUU  CUAU
   GGGU UCUUU UAA  GAUG
UAA    U     U   UU
```

mir-8196b

```
      U     U-   UU     UC
  CCCA AGAAA  AUU  CUAUG
  GGGU UCUUU  UAA  GAUGU
AA    U     UU   UU

      U     U-   UU    GUC
  CCCA AGAAA  AUU  CUAU
  GGGU UCUUU  UAA  GAUG
AA    U     UU   UU    U
```

mir-8197

```
    U        CCAC     UC
  AG GCUUUGCU    CCAAC
  UC UGGAACGA    GGUUG
AG  U        AAC-

    U        CCAC     UC
  AG GCUUUGCU    CCAAC
  UC UGGAACGA    GGUUG
AG  U        AAC-
```

mir-8198

```
          U  ---    UGUU
 UUGAACAGU UC   AAUU
 AACUUGUCA AG   UUAA
A         U  GCA    U

          UUCA      U
 UUGAACAGU    AUUUGU
 AACUUGUCA    UAGGCA
A         ----      UUAAU
```

mir-8199

```
  UCGGA       -A-   GAU
       CAAUAUC   CUG
       GUUGUAG   GAU
AUAAAAG       GAA   G

  UCGGA       A  GGAU
       CAAUAUC CU
       GUUGUAG GA
AUAAAAG       -  AGAUG
```

mir-81

```
  C         C       G GA
   GGUUUUCAC GUGAUCU A
   UCGAAAGUG UACUAGA U
UGA         C       G

  C         C       G GA
   GGUUUUCAC GUGAUCU A
   UCGAAAGUG UACUAGA U
UGA         C       G
```

mir-8200

```
                A      CC
  UGGCUCAAAUCUCC GUCAGA
  ACCGAGUUUAGAGG CAGUCU
CU              G

                A      CC
  UGGCUCAAAUCUCC GUCAGA
  ACCGAGUUUAGAGG CAGUCU
CU              G
```

mir-8201

```
                    ACCU
  UCUGGAUCGAUAAUGUAA
  AGACCUAGUUAUUACAUU
AG                  AA

                    ACCU
  UCUGGAUCGAUAAUGUAA
  AGACCUAGUUAUUACAUU
AG                  AA
```

mir-8202

```
       AA     AAA   GUG GU
     UG  CAGAA   GUC   U
     AC  GUUUU   CAG   A
CGUUA  AG     -A-   AG-

       AA     AAA   GUGUGU
     UG  CAGAA   GUC
     AC  GUUUU   CAG
CGUUA  AG     -A-   AGA
```

mir-8203

```
      C    UUCA   CA–   C
  UGAA AUCA    GGG   UUA
  GCUU UAGU    CCC   AAU
UA    A    CAAC   AUA
```

```
      C    UUCA   C   AC
  UGAA AUCA    GGG AUU
  GCUU UAGU    CCC UAA
UA    A    CAAC   A   AU
```

mir-8204

```
          A C      U     UU
   UGGUCUC C ACGCGU ACUCA
   ACCGGAG G UGCGCG UGGGU
CUU       C A      U
```

```
          A C      U     UU
   UGGUCUC C ACGCGU ACUCA
   ACCGGAG G UGCGCG UGGGU
CUU       C A      U
```

mir-8205

```
     C     UC  U
  UGG AGACU  GU GAGGCUA
  ACC UUUGA  CA CUCCGAU
GC   U     CC  –       U
```

```
     C     UC  U
  UGG AGACU  GU GAGGCUA
  ACC UUUGA  CA CUCCGAU
GC   U     CC  –       U
```

mir-8206

```
      U              UCA
  UAUA AAUGUAAUCUGAAA
  AUAU UUACAUUAGACUUU
GG    C
```

```
      U              UCA
  UAUA AAUGUAAUCUGAAA
  AUAU UUACAUUAGACUUU
GG    C
```

mir-8207

```
      C         C   UU  CA
  UUGU CUCUUUUCU UCA  UG
  AGCA GAGAAAAGA GGU  AC
AG    A         A   U–
```

```
      C         C   UUUGCA
  UUGU CUCUUUUCU UCA
  AGCA GAGAAAAGA GGU
AG    A         A   UAC
```

mir-8208

```
           CAG         UU
   UCCGCCCA   UUGAACCAA
   AGGCGGGU   GACUUGGUU
UCA        AAG
```

```
           CAG         UU
   UCCGCCCA   UUGAACCAA
   AGGCGGGU   GACUUGGUU
UCA        AAG
```

mir-8209

```
  A        AA          A
   AAACGAAG  AGAAGAAGA
   UUUGCUUC  UCUUCUUCU
CCC        CC
```

```
  A        AA          A
   AAACGAAG  AGAAGAAGA
   UUUGCUUC  UCUUCUUCU
CCC        CC
```

mir-8210

```
 UG C       C  GUG   C  AC
   C UUCUUUC UU   UCG CG
   G AAGAAAG AA   AGC GC
AAA A       A  AAA   A
```

```
 UG C       C  GUG   C  AC
   C UUCUUUC UU   UCG CG
   G AAGAAAG AA   AGC GC
AAA A       A  AAA   A
```

mir-8211

```
  A        –        UG
   CUCGAGGC CGGUGAGC  C
   GAGCUUCG GCCGCUCG  G
CAA        A        –– CA
```

```
  A        –          C
   CUCGAGGC CGGUGAGCUG
   GAGCUUCG GCCGCUCGGC
CAA        A          A
```

mir-8212

```
               AC   A  GU
  UUGCUCAAAAAUU  UUC AA
  AACGAGUUUUUGA  GAG UU
UC             CA   C
```

```
               AC   AAAGU
  UUGCUCAAAAAUU  UUC
  AACGAGUUUUUGA  GAG
UC             CA   CUU
```

mir-82

```
  C       U U       A  GA
   GGUUUUC C GUGAUCU CA
   CCGAAAG G UACUAGA GU
UGA       U C       –
```

```
  C       U U       A  GA
   GGUUUUC C GUGAUCU CA
   CCGAAAG G UACUAGA GU
UGA       U C       –
```

mir-83

```
                U  A  UGA
  ACUGAAUUUAUGUG GU CU
  UGACUUAAAUAUAC CA GA
AA              –  C  U
```

```
                –   ACUUGA
  ACUGAAUUUAUGUG UGU
  UGACUUAAAUAUAC ACG
AA              C   AU
```

mir-84

```
      G    A  U        AGA
  UGAG UAGU UG AAUAUUGU
  GCUC AUCA AC UUGUAACA
CG    A    –  U        C
```

```
      G    A  U        AGA
  UGAG UAGU UG AAUAUUGU
  GCUC AUCA AC UUGUAACA
CG    A    –  U        C
```

mir-85

```
  C           –  G    A AC
   CGAUUUUUCAA UA UUUG A
   GCUGAAAAGUU AU AAAC U
CGU           U  G    A
```

```
  C           –  G    AAAC
   CGAUUUUUCAA UA UUUG
   GCUGAAAAGUU AU AAAC
CGU           U  G    AU
```

mir-86

```
            C  UU   A   UC
   UAAGUGAAU CU  GCC CAG
   AUUCGCUUA GA  CGG GUC
CGG         –  CU   –
```

```
            C  UU   A   UC
   UAAGUGAAU CU  GCC CAG
   AUUCGCUUA GA  CGG GUC
CGG         –  CU   –
```

mir-87

```
          U     C U    A CU
   CGCCUGA ACUUU G CUCA C
   GUGGACU UGAAA C GAGU G
CGU       U     – –    –
```

```
          U     C U    ACCU
   CGCCUGA ACUUU G CUCA
   GUGGACU UGAAA C GAGU
CGU       U     – –    G
```

mir-90

```
  C   U     –    G      AC
   GGC UUCAA CGAC AUAUCA
   CCG AAGUU GUUG UAUAGU
UCC   U         U   –
```

```
  C   U     –    G      AC
   GGC UUCAA CGAC AUAUCA
   CCG AAGUU GUUG UAUAGU
UCC   U         U   –
```
